# MRS2 missense variation at Asp216 abrogates inhibitory Mg^2+^ binding, potentiating cell migration and apoptosis resistance

**DOI:** 10.1002/pro.5108

**Published:** 2024-07-11

**Authors:** Sukanthathulse Uthayabalan, Taylor Lake, Peter B. Stathopulos

**Affiliations:** ^1^ Department of Physiology and Pharmacology, Schulich School of Medicine and Dentistry University of Western Ontario London Ontario Canada

**Keywords:** apoptosis, feedback inhibition, gastric cancer, ion channels, magnesium ions (Mg^2+^), Mg^2+^ sensor, mitochondria, mitochondrial RNA splicing protein 2 (MRS2), signal transduction

## Abstract

Mitochondrial magnesium (Mg^2+^) is a crucial modulator of protein stability, enzymatic activity, ATP synthesis, and cell death. Mitochondrial RNA splicing protein 2 (MRS2) is the main Mg^2+^ channel in the inner mitochondrial membrane that mediates influx into the matrix. Recent cryo‐electron microscopy (cryo‐EM) human MRS2 structures exhibit minimal conformational changes at high and low Mg^2+^, yet the regulation of human MRS2 and orthologues by Mg^2+^ binding to analogous matrix domains has been well established. Further, a missense variation at D216 has been identified associated with malignant melanoma and MRS2 expression and activity is implicated in gastric cancer. Thus, to gain more mechanistic and functional insight into Mg^2+^ sensing by the human MRS2 matrix domain and the association with proliferative disease, we assessed the structural, biophysical, and functional effects of a D216Q mutant. We show that the D216Q mutation is sufficient to abrogate Mg^2+^‐binding and associated conformational changes including increased α‐helicity, stability, and monomerization. Further, we reveal that the MRS2 matrix domains interact with ~μM affinity, which is weakened by up to two orders of magnitude in the presence of Mg^2+^ for wild‐type but unaffected for D216Q. Finally, we demonstrate the importance of Mg^2+^ sensing by MRS2 to prevent matrix Mg^2+^ overload as HeLa cells overexpressing MRS2 show enhanced Mg^2+^ uptake, cell migration, and resistance to apoptosis while MRS2 D216Q robustly potentiates these cancer phenotypes. Collectively, our findings further define the MRS2 matrix domain as a critical Mg^2+^ sensor that undergoes conformational and assembly changes upon Mg^2+^ interactions dependent on D216 to temper matrix Mg^2+^ overload.

## 1 INTRODUCTION

Magnesium ions (Mg^2+^) play critical roles in diverse cellular processes such as protein synthesis, protein stability, modulation of enzymatic activity, as well as adenosine triphosphate (ATP) synthesis and hydrolysis (Pilchova et al., 2017; Stangherlin & O'Neill, 2018). Further, ATP‐mediated metabolism, muscle contraction and relaxation, and normal neurological functions are all Mg^2+^‐dependent functions, to name a few. Additionally, Mg^2+^ is the primary intracellular antagonist of calcium (Ca^2+^), which is an essential second messenger, initiating or regulating myriad cellular functions (Jung et al., 1990), and Mg^2+^ itself can function as a second messenger (Daw et al., 2020).

Total intracellular Mg^2+^ content ranges from ~17 to 30 mM; however, most Mg^2+^ is bound to proteins and negatively charged molecules such as ATP, resulting in free Mg^2+^ concentrations ranging between 0.5 and 1.2 mM (Jung et al., 1990; Rutter et al., 1990). In humans, cellular and subcellular Mg^2+^ concentrations are regulated by a toolkit of transporters and exchangers. Transient receptor potential melastatin type 6 (TRPM6) and TRPM7 form channels on the plasma membrane (PM) that mediate influx of Mg^2+^ (Schlingmann et al., 2007; Touyz, 2008). MagT1 has been found on the PM, regulating Mg^2+^ influx, and on the endoplasmic reticulum (ER) as part of the oligosacchyltransferase complex (Blommaert et al., 2019; Matsuda‐Lennikov et al., 2019). The nonimprinted in Prader–Willi/Angelman (NIPA) family of Mg^2+^ transporters, found on endosomes and the PM, also mediate Mg^2+^ influx (Butler et al., 2022). The cyclin and cystathionine‐β‐synthase (CBS) domain divalent metal cation transporter mediator (CNNM) family on the PM play a balancing role, likely functioning to extrude Mg^2+^ (Giménez‐Mascarell et al., 2019). Similarly, the solute carrier family 41 (SLC41) member proteins are found on the PM (SLC41A1) and inner mitochondrial membrane (IMM) (SLC41A3), functioning to efflux Mg^2+^ from the mitochondria and out of the cell in a Na^+^‐dependent manner (i.e., Na^+^/Mg^2+^ exchange) (Mastrototaro et al., 2016). Membrane Mg^2+^ transporters‐1 and ‐2 (MMgT1 and MMgT2) localized in the Golgi contain a single transmembrane (TM) domain and most probably regulate other Mg^2+^ channels rather than directly mediating Mg^2+^ flux (Goytain & Quamme, 2008). We recently identified transmembrane protein 94 (TMEM94) as the first P‐type Mg^2+^ ATPase involved in moving Mg^2+^ into the ER via a primary active transport mechanism (Vishnu et al., 2024).

Besides the ER, mitochondria represent a major sink for intracellular Mg^2+^ due to the enormous driving force of the highly negative mitochondrial membrane potential. Indeed, Mg^2+^ has a profound impact on the metabolic state through stimulatory effects on isocitrate dehydrogenase (Willson & Tipton, 1981), 2‐oxoglutarate dehydrogenase complex (Panov & Scarpa, 1996) and pyruvate dehydrogenase (PDH) phosphatase (Thomas et al., 1986), and Mg^2+^ is a known activator of the *F*_0_/*F*_1_‐ATPase (Galkin & Syroeshkin, 1999; Syroeshkin et al., 1999). Thus, cells have evolved mitochondrial RNA splicing protein‐2 (MRS2) on the IMM to carefully regulate the uptake of Mg^2+^ into the matrix (Daw et al., 2020). Human MRS2 belongs to the large heterogeneous CorA/Mrs2/Alr1 protein superfamily of Mg^2+^ transporters (Daw et al., 2020; Knoop et al., 2005; Palombo et al., 2013). Mrs2 KO bone marrow stem cells exhibit decreased respiration and ATP production (Lin et al., 2022) but this effect may be cell‐type specific (Daw et al., 2020). MRS2 overexpression in human embryonic kidney (HEK) cells increases mitochondrial Mg^2+^ flux and total cellular Mg^2+^, attenuating apoptosis (Merolle et al., 2018). Further, MRS2 is upregulated in multidrug resistant (MDR) gastric cancer (GC) cells, and the higher MRS2 activity is linked with increased resistance to apoptosis (Rawla & Barsouk, 2019).

Given the importance of matrix Mg^2+^ to mitochondrial function, it is not surprising that MRS2 has evolved Mg^2+^‐dependent autoregulation mechanisms. MRS2 contains a large amino terminal domain (NTD) localized in the matrix, two TM domains and a small carboxyl (C)‐terminal domain, also oriented in the matrix. We previously showed that Mg^2+^ binding to D216 and D220, termed here Mg^2+^‐inh (i.e., inhibitory Mg^2+^ binding site), of the MRS2‐NTD leads to channel inhibition through a mechanism involving increased secondary structure and NTD disassembly. Remarkably, the D216A/D220A double mutation profoundly enhances mitochondrial Mg^2+^ uptake (Uthayabalan et al., 2023). However, recent human MRS2 cryoelectron microscopy (cryo‐EM) structures determined in the presence and absence of Mg^2+^ have offered limited insights into Mg^2+^‐dependent feedback regulation given the conformations with and without Mg^2+^ are nearly identical (He et al., 2023; Lai et al., 2023; Li et al., 2023). Nevertheless, four Mg^2+^ binding sites have been identified in the highest resolution cryo‐EM structure with one of the Mg^2+^ interactions involving residues across two separate protomers (i.e., E261 from protomer A and E293 and E297 from protomer B) (Li et al., 2023). Interestingly, a missense mutation at D216 has been discovered in a malignant melanoma tissue sample (Van Allen et al., 2015) involving one of the residues important for negative feedback regulation.

Here, to gain further mechanistic insights into the negative feedback regulation of MRS2 activity and relationship between MRS2 function and hallmarks of cancer, we created a D216Q mutation and assessed the Mg^2+^ sensing ability of MRS2‐NTD. We find that while D216Q robustly abrogates Mg^2+^‐dependent increases in α‐helicity, stability, and hydrodynamic radius (*R*_h_), an E261Q mutant designed to disrupt the Mg^2+^‐dependent bridging between protomers based on cryo‐EM data shows wild‐type sensitivity to the cation. Further, we show that Mg^2+^ weakens the association between MRS2‐NTD monomers by as much as two orders of magnitude, with the D216Q mutant not only suppressing Mg^2+^ binding affinity but also permitting stable MRS2‐NTD associations even in the presence of high mM Mg^2+^. Consistent with the profound effect on MRS2‐NTD biophysical properties, Mg^2+^ induces a distinct conformation as indicated by Cys‐crosslinking that can be blocked by the D216Q but not E261Q mutation. Finally, we show that overexpression of MRS2‐D216Q intensifies mitochondrial Mg^2+^‐uptake, accelerates cell migration and attenuates cell death after apoptotic challenge. Collectively, our work demonstrates D216 is a critical determinant of Mg^2+^‐dependent feedback regulation in MRS2 where mutation may contribute to key hallmarks of cancer.

## 2 RESULTS

**FIGURE 1:**
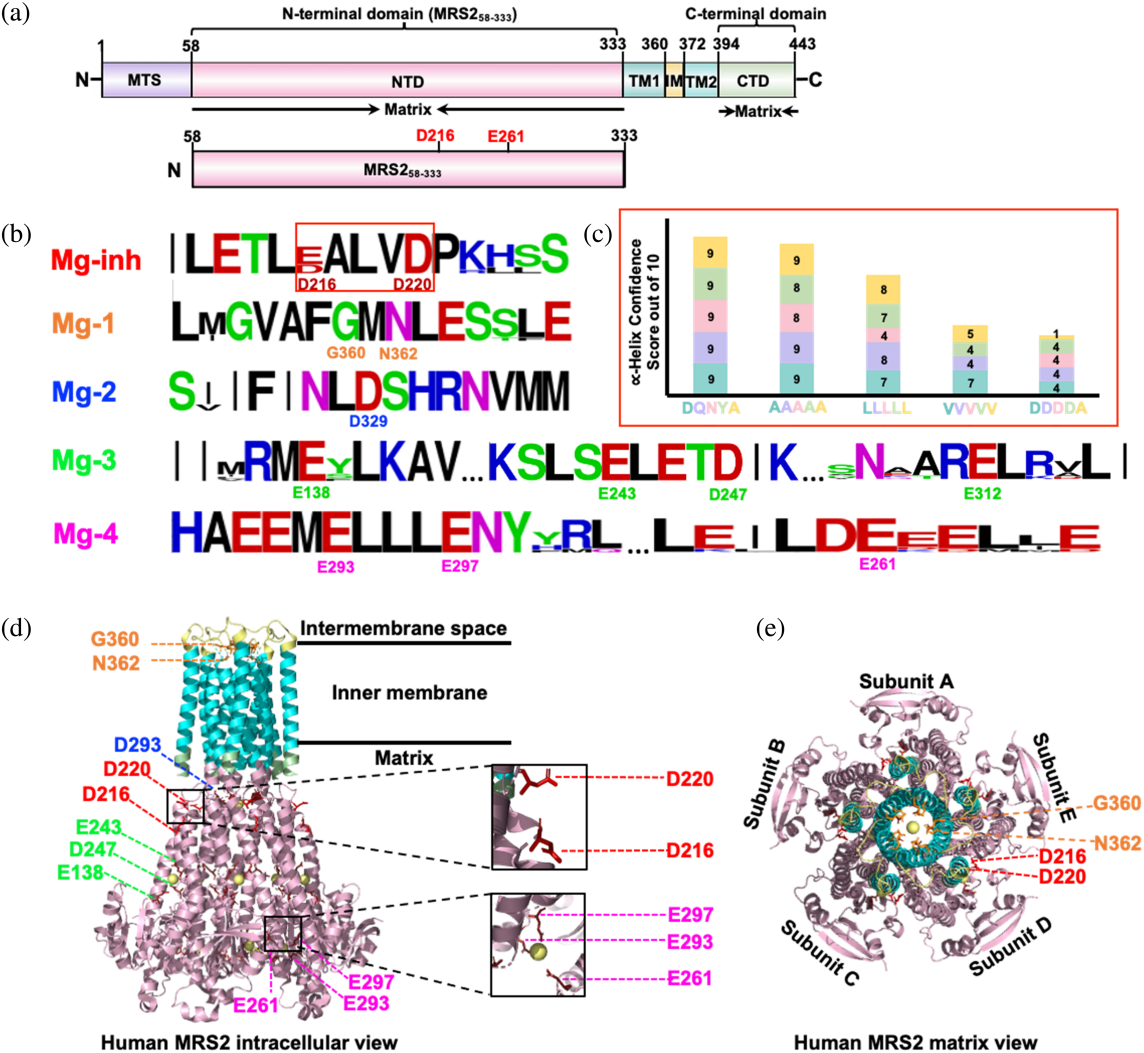
Domain architecture of human MRS2 and sequence logo of human MRS2 Mg^2+^ binding sites. (a) Domain architecture of human MRS2. The relative locations of the mitochondrial targeting sequence (MTS, purple), amino terminal domain (NTD, pink), transmembrane 1, 2 (TM1/2, cyan), intermembrane space (IM, yellow), and C‐terminal domain (CTD, green) are shown. The residue ranges are shown above and below each domain, labeled based on NCBI: NP_065713.1. Below, the N‐terminal domain (NTD, pink) construct is shown relative to the entire pre‐protein. Mutation sites assessed in the present study are indicated above the domain. (b) Sequence logos of Mg^2+^ inhibition (Mg‐inh) site and Mg^2+^ binding sites are shown. Sequence logo output was derived using WebLogo from the Clustal Omega alignment of sequences for *Homo sapiens* (NCBI Reference Sequence: NP_065713.1), *Bos taurus* (NP_001095373.1), *Canis lupus* (XP_038302408.1), *Oryctolagus cuniculus* (XP_002714204.2), *Mus musculus* (NP_001013407.2), *Gallus gallus* (XP_040519067.1) *Ornithorhynchus anatinus* (XP_028909352.1), *Zootoca vivipara* (XP_060133926.1), *Rana temporaria* (XP_040209734.1), *Danio rerio* (XP_693621.5), with defaults (Sievers et al., 2011). (c) Psipred confidence scores are shown for DALVD (blue), QALVD (purple), NALVD (pink), YALVD (green), and AALVA (yellow) MRS2 mutations. (d) Intracellular view of the cryo‐EM human MRS2 structure (8IP3). The M‐1 (orange sticks), M‐2 (dark blue sticks), M‐3 (green sticks), M‐4 (magenta sticks) sites are indicated. The DALVD Mg‐inh is shown (red sticks). The NTD (pink), TM1/2 (cyan), intermembrane space loops (yellow), and CTD (green) are colored in accordance to the domain architecture. (e) Matrix view of the cryo‐EM human MRS2 structure (8IP3). The GMN motif (orange sticks) and DALVD Mg‐inh (red sticks) are shown.

We previously identified an inhibitory Mg^2+^ binding site (Mg^2+^‐inh) on human MRS2 localized at near the D_216_ALVD_220_ residue range of the NTD (Figure 1b and Figure S1; Sievers et al., 2011). A D216A/D220A mutation abrogated Mg^2+^‐binding induced increased α‐helicity and stability as well as MRS2‐NTD dimer dissociation, but dramatically enhanced mitochondrial Mg^2+^ uptake (He et al., 2023). A review of the Genome Aggregation Database (gnomAD) revealed D216N and D216Y as missense mutations present in an aggregate of ~195,000 sequenced individuals (Gudmundsson et al., 2022). The D216N mutation was also identified as a somatic mutation in malignant melanoma (Van Allen et al., 2015). Given the increased MRS2‐NTD α‐helicity associated with Mg^2+^ binding at the DALVD region, we used PSIPRED to predict whether the D216N or D216Y alter the propensity for this secondary structure (Buchan & Jones, 2019). Both D216N and D216Y diminished confidence in α‐helicity within the DALVD region; in contrast, we found that a D216Q mutation maintained a closer wild‐type‐like helical propensity in the DALVD stretch, taken as the sum of the confidence scores for each of the five residues (Figure 1c). Thus, to investigate the impact of variation at the D216 site while maintaining a comparable α‐helical propensity as the wild‐type MRS2‐NTD, the D216Q mutation was generated.

### 2.1 The D216Q mutation prevents Mg^2+^‐induced MRS2‐NTD monomerization

**FIGURE 2:**
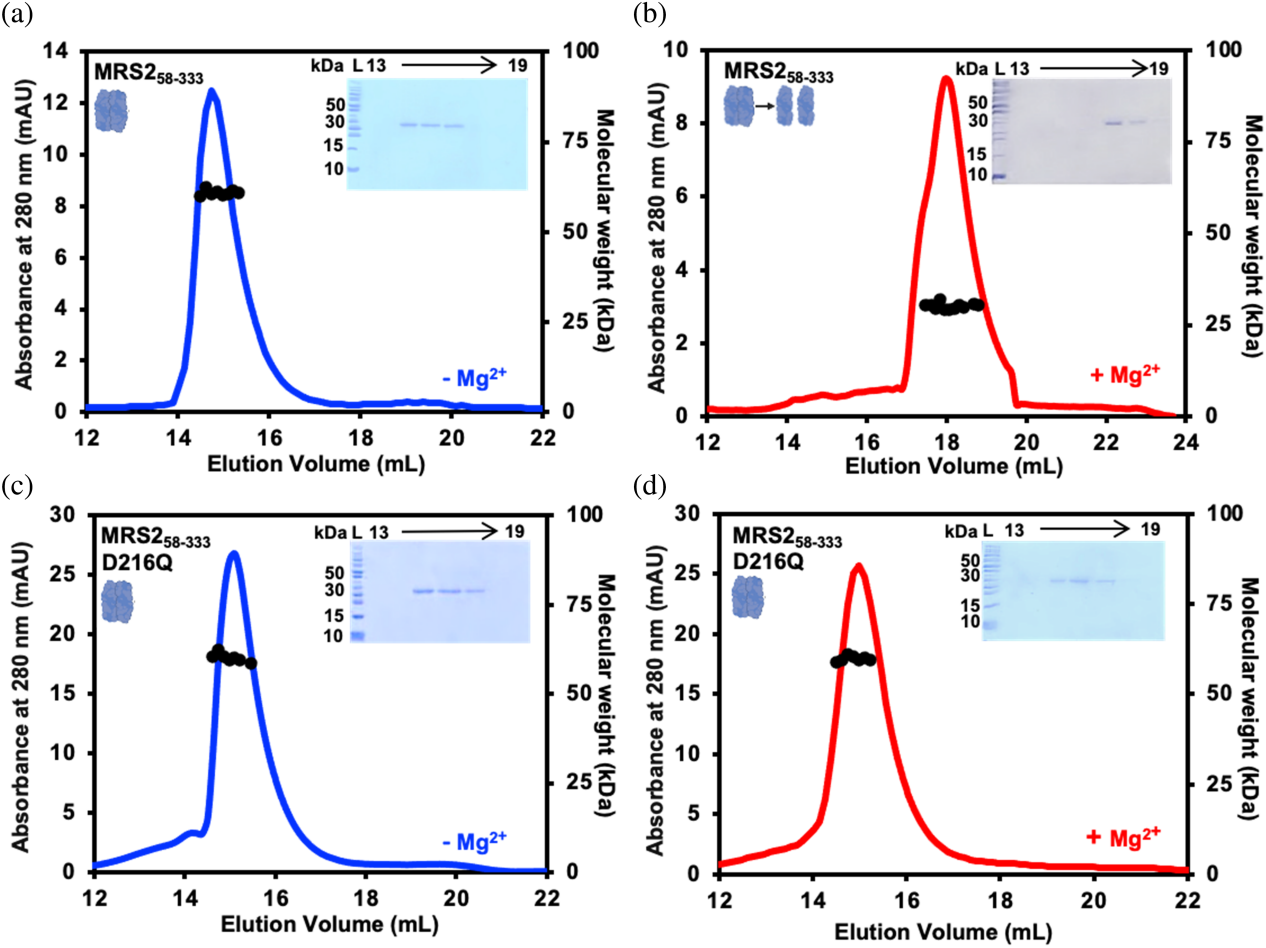
Quaternary structure of MRS2_58‐333_ (NTD) and MRS2_58‐333_‐D216Q. SEC‐MALS analysis of MRS2_58‐333_ injected at 2.5 mg/mL in (a) the absence and (b) the presence of 5 mM MgCl_2_. SEC‐MALS analysis of MRS2_58‐333_‐D216Q injected at 2.5 mg/mL in (c) the absence and (d) the presence of 5 mM MgCl_2_. In (*A, B, C, D*), MALS‐determined molecular weights are shown through the elution peaks (black circles), left insets show Coomassie blue‐stained SDS‐PAGE gels of the elution fractions from the 2.5 mg/mL injections, right insets depict the dimerization state of the protein and divalent cation‐free and supplemented chromatograms are colored blue and red, respectively. Elution volumes are indicated at top and ladder (L) molecular weights at left of the gels. Data are representative of *n* = 3 separate injections from three protein preparations and were acquired using an S200 10/300 GL column in 20 mM Tris, 150 mM NaCl, and 1 mM DTT, pH 8.0, 10°C.

The human MRS2‐NTD consisting of residues 58‐333 (MRS2_58‐333_; NCBI Reference Sequence NP_065713.1) has a theoretical monomer molecular weight of 32.5 kDa (including cloning artifacts). Size‐exclusion chromatography (SEC) with inline multiangle light scattering (MALS) analysis of recombinantly expressed and purified protein revealed a dimeric molecular weight of 57.6 ± 2.4 kDa in buffer nominally free of Mg^2+^ (Figure 2a). The introduction of 5 mM MgCl_2_ into the elution buffer‐induced robust monomerization with the mean measured molecular weight shifting to 34.0 ± 2.3 kDa (Figure 2b) concomitant with a shift to later elution volume (Table S1). Next, we assessed the quaternary structure of MRS2‐NTD‐D216Q with and without Mg^2+^. In the absence of Mg^2+^, the D216Q mutant protein eluted as a as a dimer with a molecular weight of 55.1 ± 3.8 kDa at 2.5 mg/mL, similar to wild‐type (Figure 2c). However, in contrast to wild‐type, the SEC‐MALS–determined molecular weight of D216Q remained dimeric (55.5 ± 1.8 kDa) even after the supplementation of the elution buffer with 5 mM MgCl_2_ (Figure 2d) with no change in elution volume (Table S1).

### 2.2 Mg^2+^weakens the self‐association of the MRS2‐NTD by up to ~100‐fold

**FIGURE 3:**
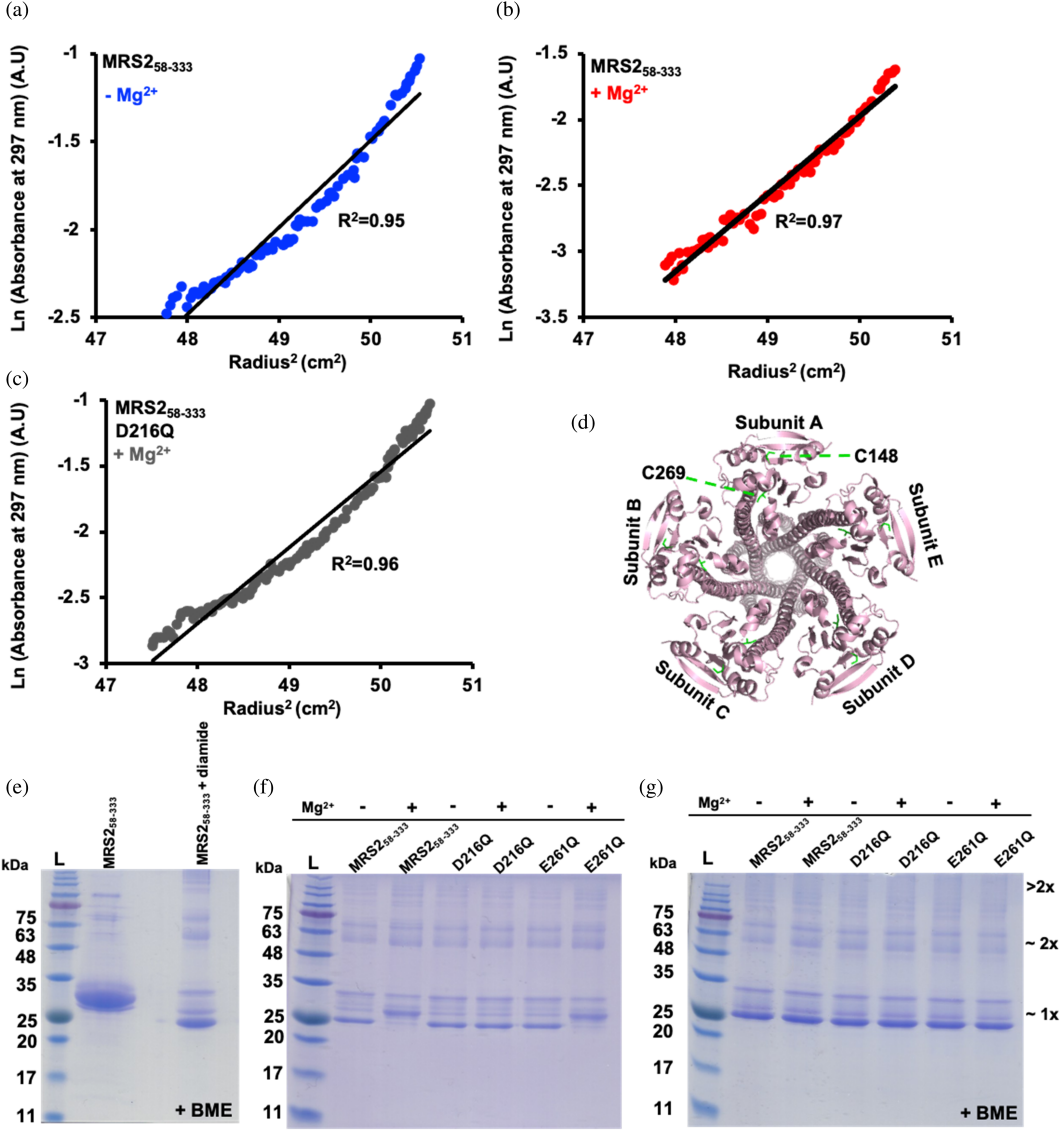
Equilibrium ultracentrifugation assessment and cysteine cross‐linking of MRS2_58‐333_, MRS2_58‐333_‐D216Q, and MRS2_58‐333_‐E261Q. (a) Ln(absorbance) versus radius^2^ plots for MRS2_58‐333_ highlighting nonlinearity and high self‐association in the absence of 5 mM MgCl_2_ (blue circles). (b) Ln(absorbance) versus radius^2^ plots for MRS2_58‐333_ highlighting increased linearity and decreased self‐association in the presence of 5 mM MgCl_2_ (red circles). (c) Ln(absorbance) versus radius^2^ plots for MRS2_58‐333_‐D216Q highlighting maintained nonlinearity and high self‐association in the presence of 5 mM MgCl_2_ (gray circles). (d) Matrix view of the human MRS2 cryo‐EM structure (8IP3), showing all cysteine residues (green). (e) Reducing (+BME) Coomassie‐blue stained SDS‐PAGE of MRS2_58‐333_ in the absence and presence of diamide highlighting irreversible migration changes to the protein. (f) Non‐reducing Coomassie‐blue stained SDS‐PAGE of diamide‐treated MRS2_58‐333_, MRS2_58‐333_‐D216Q and MRS2_58‐333_‐E261Q in the presence (+) and absence (−) of 5 mM MgCl_2_. (g) Reducing (+BME) Coomassie‐blue stained SDS‐PAGE of diamide‐treated MRS2_58‐333_, MRS2_58‐333_‐D216Q and MRS2_58‐333_‐E261Q in the presence (+) and absence (−) of 5 mM MgCl_2_. The approximate location of monomer (~1×), dimer (~2×) and oligomer (>2×) bands are indicated at right. 5 mM BME was used for reducing conditions.

To assess the strength of the MRS2‐NTD homomeric associations in the absence of the ~20‐fold column dilution that occurs during SEC‐MALS and to quantify equilibrium dissociation constants (*K*_d_), we next performed analytical ultracentrifugation (AUC) experiments. Consistent with the SEC‐MALS performed at ~10°C, we found wild‐type MRS2‐NTD self‐associates in the absence of Mg^2+^, as indicated by the strong nonlinearity of a plot of ln(absorbance) versus radius^2^ during sedimentation equilibrium experiments at 20°C (Figure 3a). In the presence of 5 mM MgCl_2_, we observed an increase in the linearity consistent with disrupted self‐association (Figure 3b); however, some nonlinearity indicative of weak association persists. Indeed, global fitting of equilibrium data collected with no Mg^2+^ at 1.2, 0.6, and 0.3 mg/mL to a single ideal species revealed molecular weights of 56, 51, and 54 kDa, respectively, indicative of fast exchange dimer formation and inline with the SEC‐MALS data (Table 1). The presence of 5 mM MgCl_2_ shifted the equilibrium toward monomer with molecular weights of 48, 35, and 32 kDa at 1.2, 0.6, and 0.3 mg/mL, respectively (Table 1). The *K*_d_s for the monomer‐dimer equilibrium were also extracted using global fits at the three protein concentrations with and without Mg^2+^. Remarkably, the presence of MgCl_2_ increased the dimerization *K*_d_s (i.e., decreased the affinities) from 16, 24 and 17 μM to 55, 146, and 1761 μM for the 1.2, 0.6, and 0.3 mg/mL datasets, respectively. Notably, AUC experiments performed with the MRS2‐NTD‐D216Q mutant in the presence of 5 mM Mg^2+^ revealed a maintained high nonlinearity in the ln(absorbance) versus radius^2^ plot, analogous to wild‐type MRS2‐NTD in the absence of the divalent cation (Figure 3a,c). Further, not only were the dimer molecular weights maintained for MRS2‐NTD‐D216Q in the presence of 5 mM MgCl_2_ (i.e., ~53, 58, and 64 kDa) but the dimerization *K*_d_s were similar to wild‐type MRS2‐NTD determined in the absence of Mg^2+^ (i.e., 34, 22, and 11 μM) at 1.2, 0.6, 0.3 mg/mL, respectively (Table 1).

**TABLE 1:** Molecular weights and dimerization affinities extracted from AUC data.

Protein (MRS2_58‐333_)	Concentration (mg/mL)	Theoretical MW (Da)	MW (Da)^a^	MW error (Da)	Stoichiometry^b^	*K*_d,dimer_ (μM)^c^	*R* ^2^
WT	1.250	32,504	56,154	477	1.7	16.43	0.9955
WT	0.625	32,504	50,677	1373	1.6	24.33	0.9675
WT	0.313	32,504	53,964	3376	1.7	17.36	0.8297
WT + Mg^2+^	1.250	32,504	47,731	942	1.5	55.38	0.9850
WT + Mg^2+^	0.625	32,504	35,093	2144	1.1	146.17	0.9194
WT + Mg^2+^	0.313	32,504	31,534	4154	1.0	1760.97	0.9281
D216Q + Mg^2+^	1.250	32,517	52,987	502	1.6	33.62	0.9938
D216Q + Mg^2+^	0.625	32,517	57,970	1331	1.8	22.16	0.9755
D216Q + Mg^2+^	0.313	32,517	64,100	2351	2.0	11.18	0.9622

*Note*: Note that “+ Mg^2+^” experiments included 5 mM MgCl_2_ in the experimental buffer (20 mM Tris, 150 mM NaCl, 1 mM DTT, pH 8).

^a^ Globally fitted single ideal molecular weights from data acquired at 8000, 12,000, 16,000, and 20,000 rpm.

^b^ Fitted molecular weight/theoretical monomeric molecular weight.

^c^ Globally fitted monomer‐dimer equilibrium dissociation constant from data acquired at 8000, 12,000, 16,000, and 20,000 rpm.

Collectively, the SEC‐MALS and AUC data demonstrate that the MRS2‐NTD self‐association is profoundly affected by Mg^2+^, with mM concentrations of Mg^2+^ decreasing the dimerization *K*_d_ by up to ~100 fold at the lowest protein concentration evaluated. Further, this Mg^2+^ sensitivity can be abrogated by a simple D216Q point mutation, restoring dimerization *K*_d_s to wild‐type like affinities.

### 2.3 Mg^2+^ perturbs MRS2‐NTD conformation and/or dynamics

Recent cryo‐EM structures of human MRS2 in the presence and absence of high Mg^2+^ reveal almost identical conformations [i.e., all atom root mean square deviation (RMSD) of <0.2 Å for 8IP3.pdb determined with 20 mM MgCl_2_ aligned with 8IP4.pdb determined with 5 mM EDTA and no MgCl_2_; 8IP3, 8IP4 are the highest resolution available human MRS2 structures]. Yet, these cryo‐EM data identify four Mg^2+^ binding sites (Figure 1b) (Li et al., 2023). The human MRS2‐NTD encodes two Cys (i.e., C148 and C269). The thiols of C148 and C269 are too distant for intermolecular disulfide formation based on the static conformations and assemblies of the cryo‐EM structures (Figure 3d). Nevertheless, we sought to determine whether these Cys could serve as reliable indicators of Mg^2+^‐dependent conformational changes and/or dynamics through Cys‐crosslinking experiments. We found that treatment with 3.2 mM diamide oxidant caused several changes to the banding pattern on SDS‐PAGE gels. First, a band with increased migration compared with the untreated ~32.2 kDa MRS2‐NTD monomer band appeared close to ~25 kDa; second, several higher order bands >75 kDa appeared; third, two bands appeared close to the ~63 kDa marker (Figure 3e). Addition of 12.5 mM beta‐mercaptoethanol (BME) reducing agent did not restore the banding pattern to that observed in the absence of diamide, indicating an irreversibility associated with diamide incubation and ambiguating whether the migration changes are Cys‐mediated (Figure 3e). Nevertheless, diamide treatment of MRS2‐NTD in the presence of 5 mM MgCl_2_ resulted in a decreased migration of the frontmost band, indicative of a distinct conformation compared with the absence of Mg^2+^ (Figure 3f). Further, the migration was restored with BME addition, suggesting an intramolecularly Cys‐crosslinked protein (Figure 3g). Remarkably, the D216Q mutation prevented the Mg^2+^‐dependent conformational change and Cys‐crosslinking as the banding pattern both with and without Mg^2+^ were similar to wild‐type MRS2‐NTD in the absence of Mg^2+^ (Figure 3f). The cryo‐EM elucidated Mg‐4 binding site involving E261 from one subunit and E293 and E297 from an adjacent subunit, collectively bridge the same Mg^2+^ ion with the shortest coordination distances, implying the tightest binding (Figure 1b,d,e). Thus, we also evaluated Cys‐crosslinking of MRS2‐NTD‐E261Q, finding Mg^2+^‐sensitive Cys‐crosslinking similar to wild‐type MRS2‐NTD (Figure 3f,g).

Together, these diamide experiments indicate that Mg^2+^ induces conformational changes in the MRS2‐NTD that are dependent on D216. Further, consistent with cryo‐EM structures that exhibit no conformational differences in the presence and absence of Mg^2+^ coordinated between subunits, the E261Q mutant showed wild‐type like conformational sensitivity to Mg^2+^.

### 2.4 The D216Q mutation precludes Mg^2+^‐induced disassembly of MRS2‐NTD higher order oligomers

**FIGURE 4:**
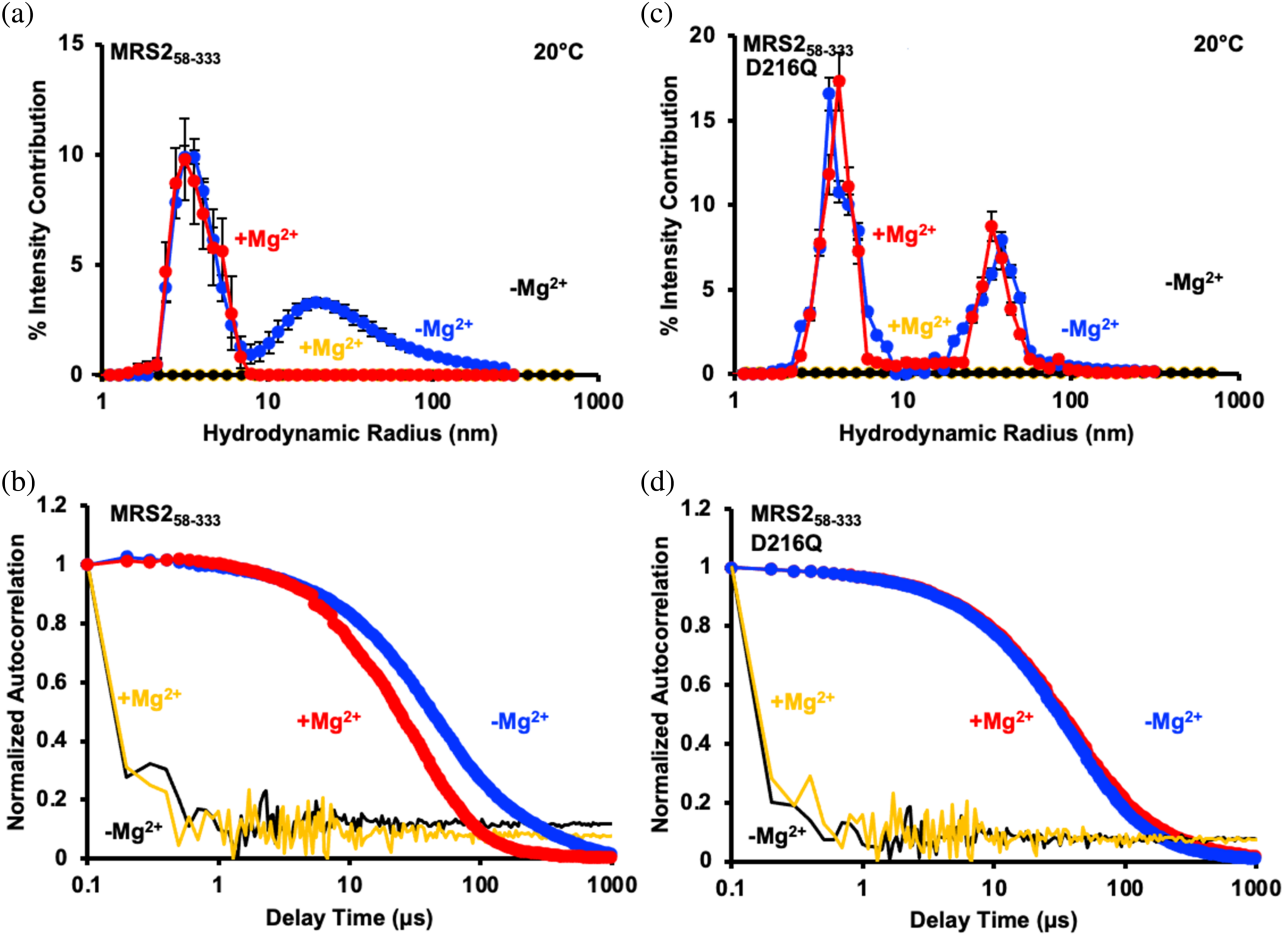
Higher order oligomerization of MRS2_58‐333_ and MRS2_58‐333_‐D216Q. (a) Distributions of hydrodynamic radii (*R*_h_) from the regularization deconvolution of the autocorrelation functions for MRS2_58‐333_ in the presence (red circles) and absence of 5 mM MgCl_2_ (blue circles). (b) Autocorrelation functions for MRS2_58‐333_ in the presence (red circles) and absence of 5 mM MgCl_2_ (blue circles) in comparison to buffer control data with (yellow line) and without (black line) 5 mM MgCl_2_. (c) Distributions of *R*_h_ from the regularization deconvolution of the autocorrelation functions for MRS2_58−333_‐D216Q in the presence (red circles) and absence of 5 mM MgCl_2_ (blue circles). (d) Autocorrelation functions for MRS2_58‐333_‐D216Q in the presence (red circles) and absence of 5 mM MgCl_2_ (blue circles) in comparison to buffer control data with (yellow line) and without (black line) 5 mM MgCl_2_. Shifts in the autocorrelation functions are representative, while deconvoluted *R*_h_ profiles are means ± SEM of *n* = 3 separate samples from three protein preparations. All data were acquired at 2.5 mg/mL protein in 20 mM Tris, 150 mM NaCl, and 1 mM DTT, pH 8.0, 20°C.

While the SEC‐MALS, AUC, and crosslinking data primarily reported on the monomer‐dimer equilibria and conformational changes, we previously demonstrated that MRS2‐NTD forms higher order oligomers sensitive to Mg^2+^ (Uthayabalan et al., 2023). Hence, we next used dynamic light scattering (DLS) to assess how D216Q effects oligomer formation. In the absence of Mg^2+^, a bimodal distribution of hydrodynamic radii (*R*_h_) was observed for wild‐type MRS2‐NTD centered at ∼4 and 30 nm (Figure 4a). The addition of 5 mM MgCl_2_ to the wild‐type sample eliminated the larger sizes, leaving the distribution centered at ~4 nm (Figure 4a). The loss of larger *R*_h_ was evident qualitatively from the earlier decays in the autocorrelation functions when compared with divalent cation‐free condition (Figure 4b). Similar to the wild‐type MRS2‐NTD sample, a bimodal distribution of *R*_h_ centered at ∼4 and 40 nm was observed for MRS2‐NTD‐D216Q in the absence of Mg^2+^. However, the addition of 5 mM MgCl_2_ did not affect the bimodal distribution, as the *R*_h_ remained centered at ~4 and 40 nm (Figure 4c). Congruently, the autocorrelation functions with or without Mg^2+^ showed similar decay profiles (Figure 4d). Thus, D216 plays a central role in Mg^2+^‐induced disassembly of MRS2‐NTD higher‐order oligomers.

### 2.5 The D216Q mutation weakens Mg^2+^ binding to the MRS2‐NTD

**FIGURE 5:**
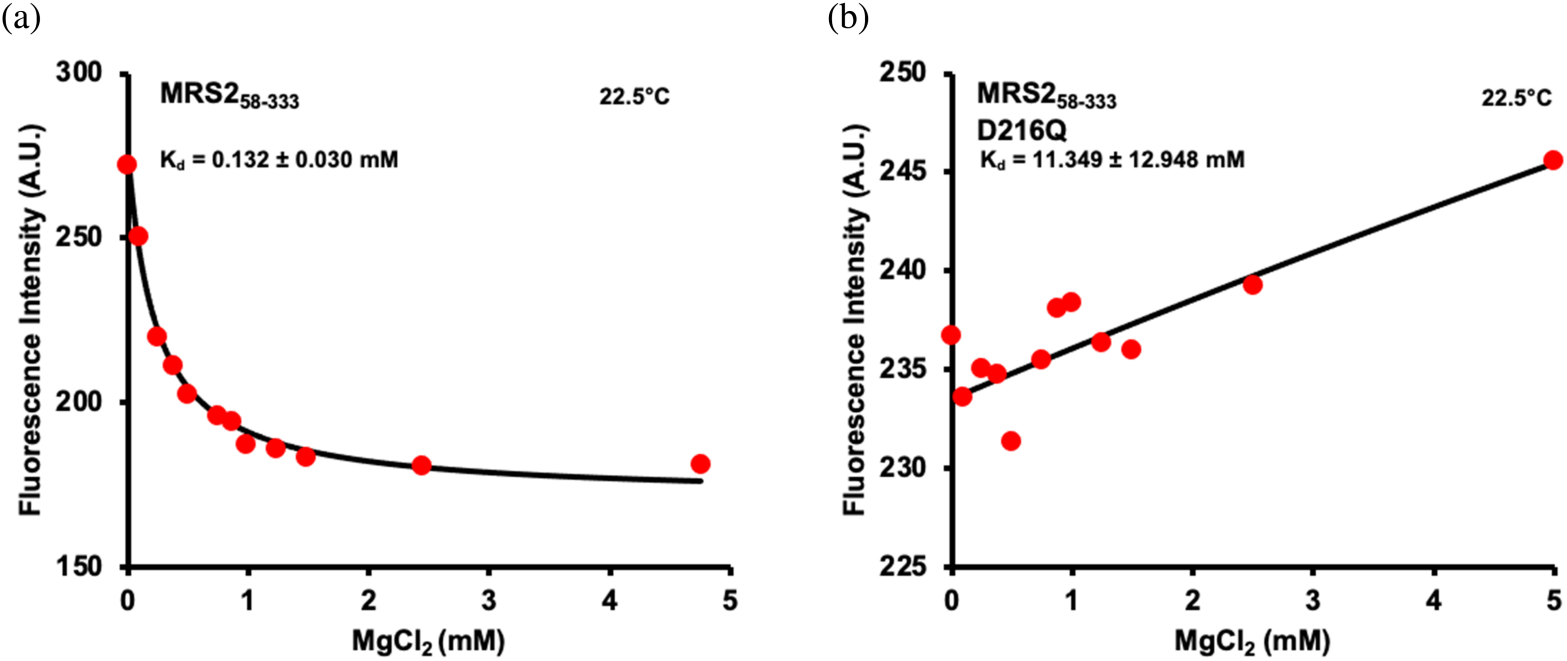
Mg^2+^ binding affinity to MRS2_58‐333_ and MRS2_58‐333_‐D216Q. (a) Changes in intrinsic fluorescence emission intensity of MRS2_58‐333_ as a function of increasing MgCl_2_ concentration. (b) Changes in intrinsic fluorescence emission intensity of MRS2_58‐333_‐D216Q as a function of increasing MgCl_2_ concentration. In (a and b) data (red circles) show intrinsic fluorescence intensities at 330 nm as a function of MgCl_2_ concentration and are representative of *n* = 3 separate experiments performed from three protein preparations. The solid black lines through the data are fits to a one‐site binding model that accounts for protein concentration. All experiments were performed with 0.1 μM protein in 20 mM Tris, 150 mM NaCl, and 1 mM DTT, pH 8.0, at 22.5°C.

Given that the D216Q mutation was sufficient to prevent the monomerization, the weakened dimerization *K*_d_, the disassembly of higher order oligomers and the conformational changes induced by incubation with Mg^2+^, we next assessed Mg^2+^ binding affinity to the wild‐type and mutant MRS2‐NTD. MRS2‐NTD contains 7 × Tyr and 3 × Trp residues, endowing the protein with strong intrinsic fluorescence. Thus, we used an excitation wavelength of 280 nm to excite both fluorophores (i.e., directly and indirectly) and acquired intrinsic fluorescence emission spectra as a function of increasing MgCl_2_ concentrations. The emission spectra exhibited a MgCl_2_ concentration‐dependent drop in intensity for the wild‐type MRS2‐NTD. Importantly, the decrease in fluorescence was saturable at near ~1 mM MgCl_2_, and fitting the decrease in fluorescence to a one‐site binding model, accounting for protein concentrations revealed an apparent Mg^2+^ interaction K_d_ of ∼0.14 ± 0.04 mM (Figure 5a). Remarkably, introducing the D216Q mutation into MRS2‐NTD completely precluded the drop in fluorescence due to Mg^2+^ addition over the same concentration range (Figure 5b). Rather, a diminutive increase in intrinsic fluorescence was observed for MRS2‐NTD‐D216Q as a function of increasing MgCl_2_, and fitting this small change to one‐site binding model revealed a *K*_d_ of ∼11.95 ± 10.05 mM (Table S2). The D216Q data not only indicate a very weak Mg^2+^ binding *K*_d_ due to the mutation but also reinforce that the change in fluorescence was not a quenching artifact by the metal ion addition. Therefore, these findings underscore the indispensability of D216 in mediating interactions with Mg^2+^.

### 2.6 The D216Q mutation stops Mg^2+^‐dependent increases in secondary structure and stability

**FIGURE 6:**
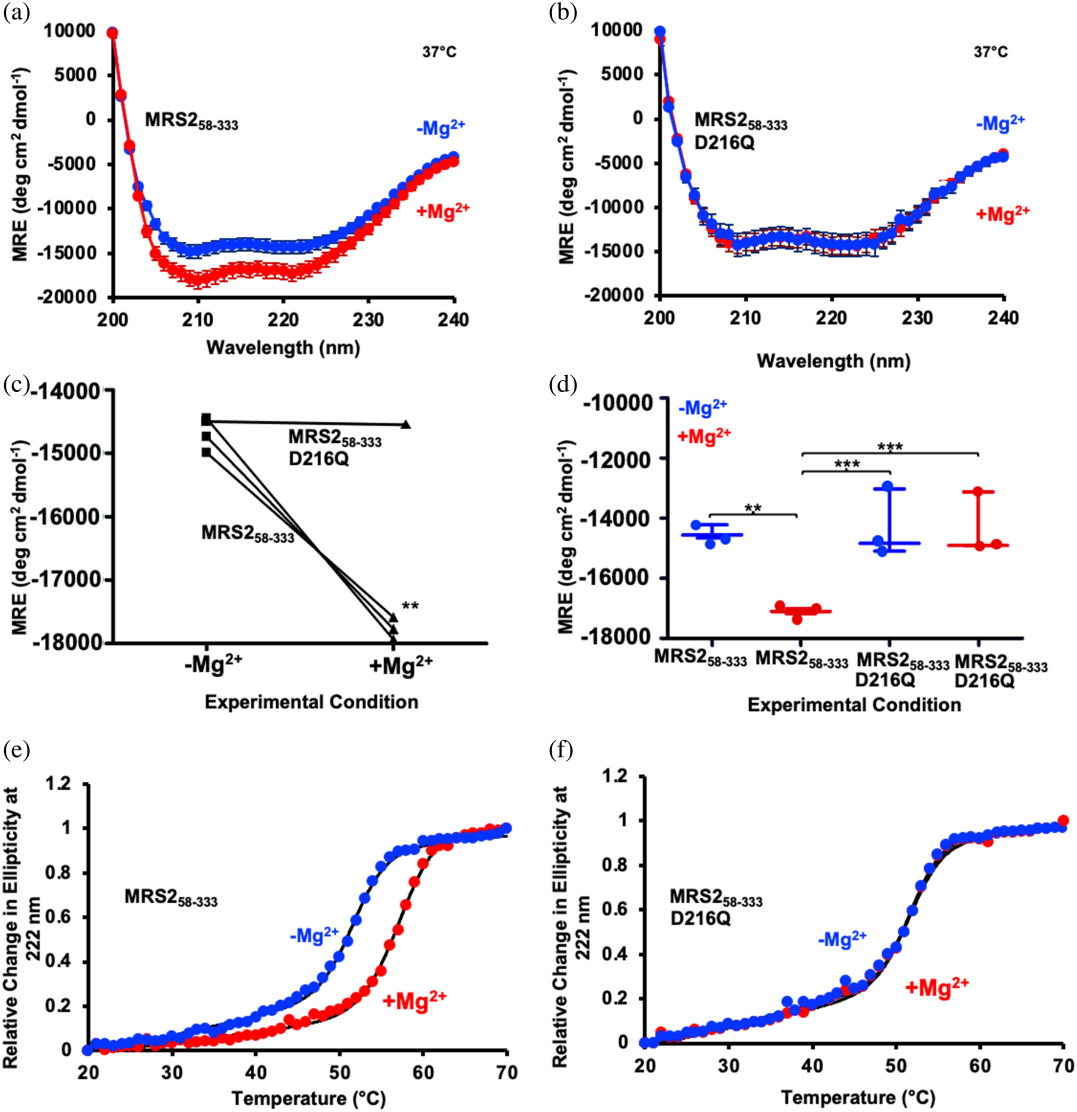
Secondary structure and thermal stability of MRS2_58‐333_ and MRS2_58‐333_‐D216Q. Far‐UV CD spectra of (a) MRS2_58‐333_ and (b) MRS2_58‐333_‐D216Q in the absence (blue) and presence (red) of 5 mM MgCl_2_. (c) Paired *t*‐test comparisons of Mg^2+^‐induced changes in mean residue ellipticity (MRE) at 222 nm for MRS2_58‐333_ and MRS2_58‐333_‐D216Q. (d) One‐way ANOVA followed by Tukey's post hoc comparison of MRE at 222 nm for MRS2_58‐333_ and MRS2_58‐333_‐D216Q in the absence (blue) and presence (red) of 5 mM MgCl_2_, where *p < 0.05, **p < 0.01, and ***p < 0.001. Thermal melts monitored as changes in MRE (222 nm) as a function of increasing temperature in the absence (blue) and presence (red) of 5 mM MgCl_2_ for (e) MRS2_58‐333_ and (f) MRS2_58‐333_‐D216Q. In (a and b), data are means ± SEM of *n* = 3 experiments with samples from three protein preparations. In (c), comparisons are of data in (a and b), where **p < 0.01. In (e and f), data (circles) are representative of *n* = 3 separate experiments with samples from three protein preparations, and solid black lines are Boltzmann sigmoidal fits through the data to extract apparent midpoints of temperature denaturation (*T*_m_). All far‐UV CD experiments were acquired with 0.5 mg/mL protein in 20 mM Tris, 150 mM NaCl, and 1 mM DTT, pH 8.0, with spectra acquired at 37°C.

Since our fluorescence and crosslinking experiments implied conformational changes associated with Mg^2+^ binding and to rule out the possibility that the D216Q mutation itself alters the structure of the MRS2‐NTD, we next assessed secondary structure of both the D216Q and wild‐type proteins. The far‐UV circular dichroism (CD) spectrum of MRS2‐NTD indicated a high level of α‐helicity with clearly defined minima at ~208 and ~222 nm, consistent with the highly α‐helical structure defined by the cryo‐EM structures. As we previously reported, addition of 5 mM MgCl_2_ directly to the cuvette quickly augmented the level of α‐helicity, as evidenced by more intense negative ellipticity at ∼208 and 222 nm (Figure 6a,c). Importantly, MRS2‐NTD‐D216Q displayed a similar level of α‐helicity as the wild‐type domain, suggesting that secondary structure folding was not perturbed by the single‐residue substitution (Figure 6b,d). Unlike wild‐type MRS2‐NTD, however, adding 5 mM MgCl_2_ directly to the cuvette did not change the shape or intensity of the MRS2‐NTD‐D216Q spectrum (Figure 6b–d).

We also evaluated thermal stability of wild‐type and D216Q MRS2‐NTD with and without Mg^2+^. Thermal melts were constructed by recording the change in far‐UV CD signal at 222 nm as a function of increasing temperature. MRS2‐NTD data acquired in the absence of Mg^2+^ exhibited a mean midpoint of temperature denaturation (*T*_m_) of 51.5 ± 0.45°C (Figure 6e and Table S1). Wild‐type protein supplemented with 5 mM MgCl_2_ was stabilized by ∼6°C as the mean *T*_m_ shifted to 57.5 ± 0.03°C (Figure 6e). This stability increase was inline with the Mg^2+^‐induced enhancement in α‐helicity observed (Figure 6a,c). In the absence of Mg^2+^, the MRS2‐NTD‐D216Q protein exhibited a mean *T*_m_ value of 51.7 ± 0.12°C, not significantly different than wild‐type MRS2‐NTD (Figure 6f and Table S1). The remarkable similarity between the wild‐type and D216Q *T*_m_ values underscores the similarities in structures. Unlike the wild‐type protein, however, the D216Q mutant *T*_m_ was unaffected by the addition of 5 mM Mg^2+^, with a mean *T*_m_ value to 52.2 ± 0.5°C (Figure 6f and Table S1).

Collectively, these spectroscopic data show that the single D216Q point mutation does not alter the secondary structure or stability of MRS2‐NTD; however, mutation of this residue renders MRS2‐NTD remarkably insensitive to Mg^2+^, highlighting the vital role of D216 in Mg^2+^ coordination.

### 2.7 The Mg‐4 E261Q mutation does not alter Mg^2+^‐dependent α‐helicity and stability changes

**FIGURE 7:**
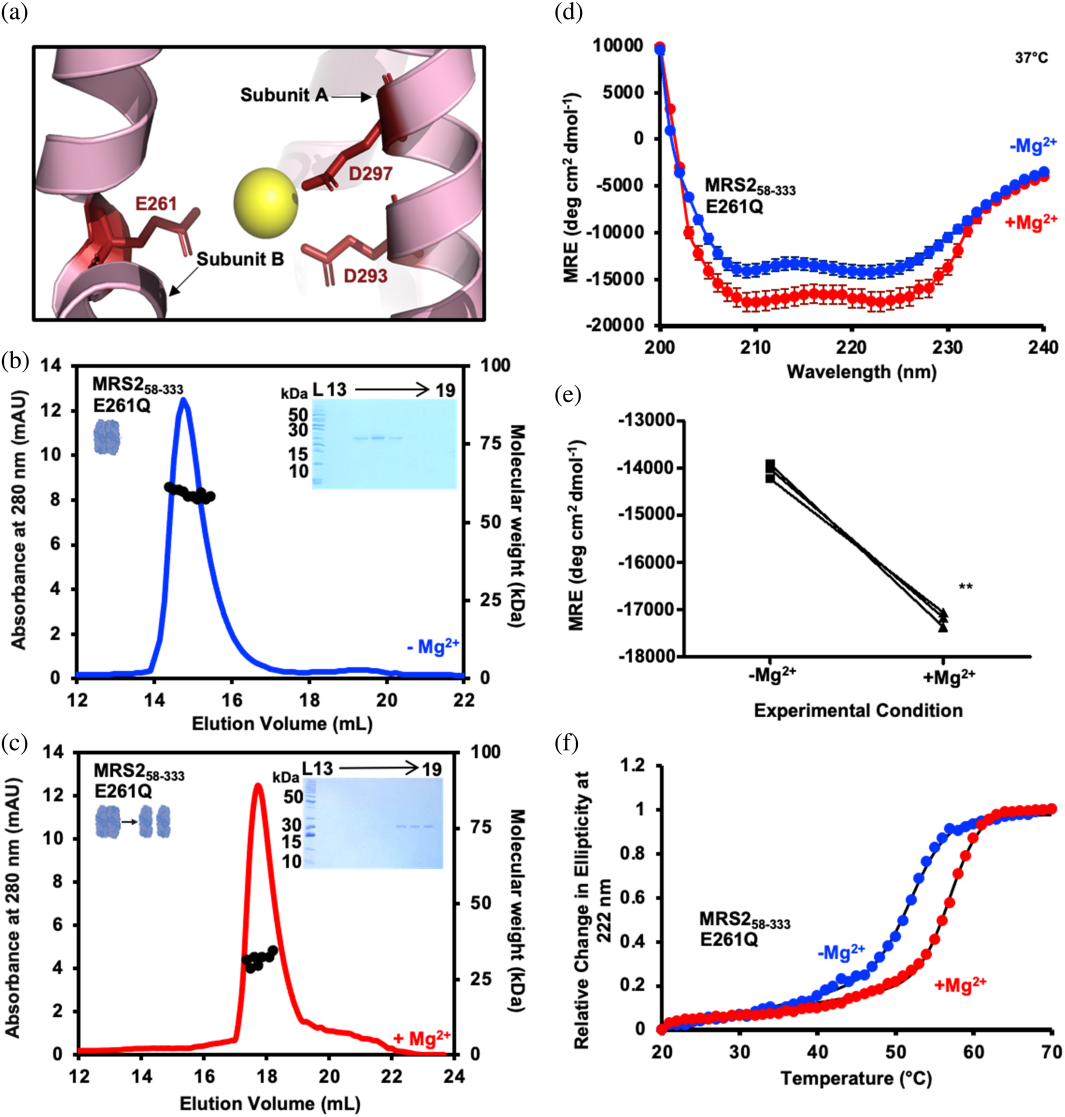
Secondary structure, thermal stability, and quaternary structure of MRS2_58‐333_‐E261Q. (a) Zoomed view of the Mg‐4 in the human cryo‐EM MRS2 structure (8IP3). Residues E261, D297, D293 are shown as red sticks. SEC‐MALS analysis of MRS2_58‐333_‐E261Q injected at 2.5 mg/mL in (b) the absence and (c) the presence of 5 mM MgCl_2_. In (b and c), MALS‐determined molecular weights are shown through the elution peaks (black circles), right insets show Coomassie blue‐stained SDS‐PAGE gels of the elution fractions from the 2.5 mg/mL injections and left insets depict the dimerization state of the protein. Elution volumes are indicated at top and ladder (L) molecular weights at left of the gels. Data are representative of *n* = 3 separate injections from three protein preparations and were acquired using an S200 10/300 GL column in 20 mM Tris, 150 mM NaCl, and 1 mM DTT, pH 8.0, 10°C. (d) Far‐UV CD spectra of MRS2_58‐333_‐E261Q in the absence (blue) and presence (red) of 5 mM MgCl_2_. (e) Paired *t*‐test comparisons of Mg^2+^‐induced changes in MRE at 222 nm for MRS2_58‐333_‐E261Q. (f) Thermal stability monitored as the change in MRE (222 nm) as a function of increasing temperature in the absence (blue) and presence (red) of 5 mM MgCl_2_ for MRS2_58‐333_‐E261Q. In (d), data are means ± SEM of *n* = 3 experiments with samples from three protein preparations. In (e), comparisons are analyses of data from (d), where *p* > 0.01. In (f), data (circles) are representative of *n* = 3 separate experiments with samples from three protein preparations, and solid black lines are Boltzmann sigmoidal fits through the data to extract *T*_m_ values. All far‐UV CD experiments were acquired with 0.5 mg/mL protein in 20 mM Tris, 150 mM NaCl, and 1 mM DTT, pH 8.0, with spectra acquired at 37°C.

Our crosslinking data indicate that mutation of the Mg‐4 Mg^2+^ binding site involving E261 from subunit B and D293 and D297 from subunit A (Figure 7a) (Li et al., 2023) does not alter Mg^2+^‐dependent Cys crosslinking within MRS2‐NTD (Figure 3f,g). Since the Cys crosslinking may be insensitive to the specific structural changes caused by Mg^2+^ binding to Mg‐4, we further explored possible modulation of MRS2‐NTD biophysical properties by evaluating SEC‐MALS, secondary structure and stability of MRS2‐NTD‐E261Q in the absence and presence of Mg^2+^. In the absence of the divalent cation, SEC‐MALS revealed that MRS2‐NTD‐E261Q elutes as a homodimer with a molecular weight of 57.3 ± 2.9 kDa when injected at 2.5 mg/mL (Figure 7b), similar to wild‐type (Figure 2a) and D216Q (Figure 2c) proteins. Following the addition of 5 mM MgCl_2_, the SEC‐MALS‐determined molecular weight of MRS2‐NTD‐E261Q shifted toward monomer (i.e., 31.8 ± 3.1 kDa) (Figure 7c), as seen for wild‐type but not D216Q (Figure 2b,d and Table S1).

Next, we acquired far‐UV CD spectra at 37°C. In the absence of Mg^2+^, MRS2‐NTD‐E261Q exhibited a high level of α‐helicity with strong negative ellipticity at ~208 and 222 nm (Figure 7d), similar to wild type and D216Q spectra acquired in the absence of Mg^2+^ (Figure 6a,b). Adding 5 mM MgCl_2_ directly to the same sample promoted a marked increase in α‐helicity, evidenced by more intense negative ellipticity at ∼208 and 222 nm (Figure 7d,e), analogous to our observations with wild‐type MRS2‐NTD but not D216Q (Figure 6a,b). Finally, thermal melts of MRS2‐NTD‐E261Q were acquired in the absence and presence of 5 mM MgCl_2_. MRS2‐NTD‐E261Q showed *T*_m_ values of 51.9 ± 0.26°C and 56.6 ± 0.29°C without and with Mg^2+^, respectively. These thermal stabilities were akin to our wild‐type measurements but distinct from the abrogation in Mg^2+^ response caused by the D216Q mutation (Figure 7f and Table S1). Thus, unlike D216Q, the E261Q mutation aimed at disrupting Mg^2+^ binding to Mg‐4 does not alter the Mg^2+^‐dependent increased α‐helicity, monomerization and enhanced stability of MRS2‐NTD.

### 2.8 Overexpression of MRS2‐D216Q augments mitochondrial Mg^2+^ uptake rates

**FIGURE 8:**
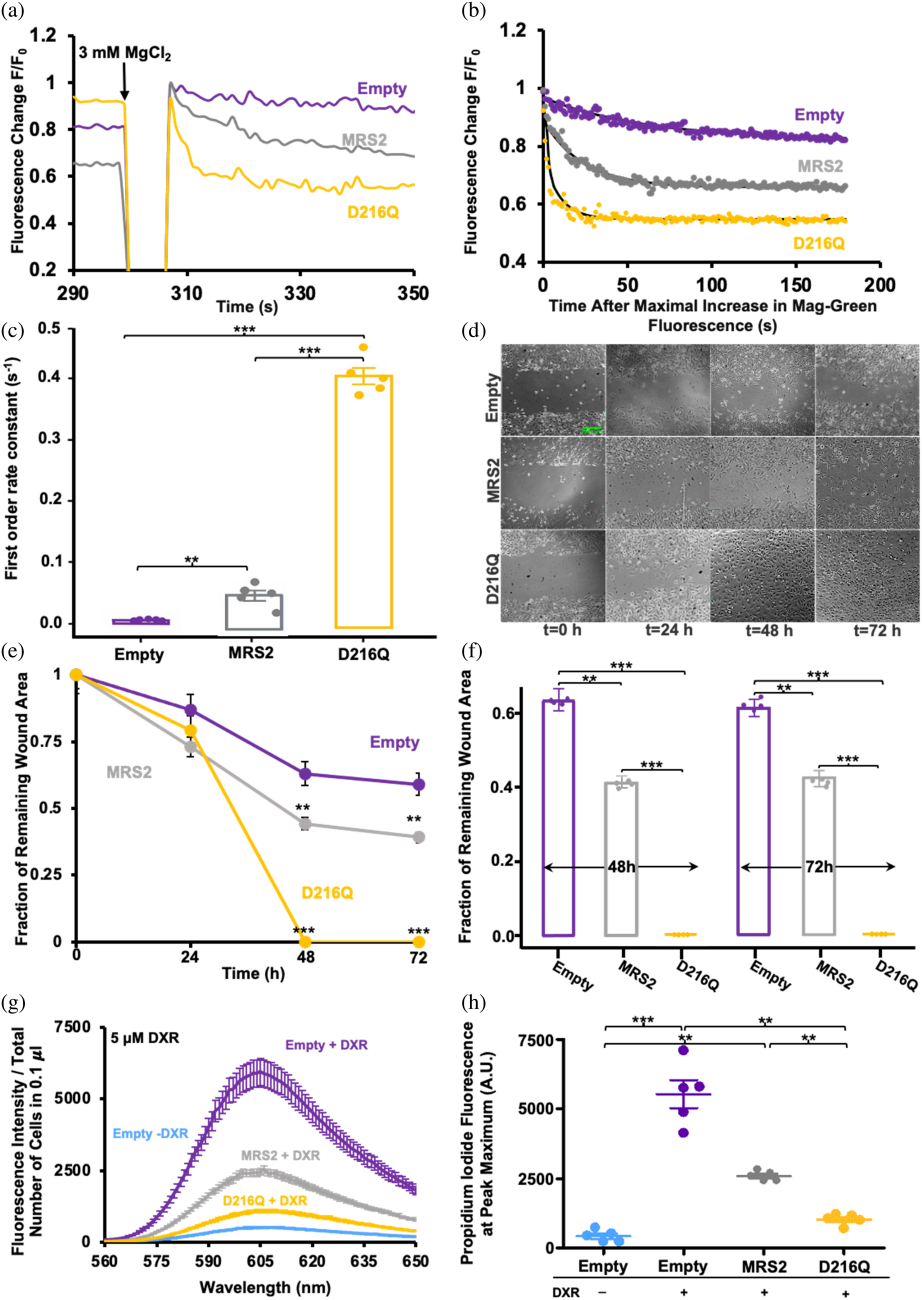
Mitochondrial Mg^2+^ uptake, cell migration and DXR‐induced cell death of HeLa cells. (a) Representative Mag‐Green fluorescence traces reporting relative extramitochondrial Mg^2+^ before and after 3 mM MgCl_2_ addback to the extracellular bath at 300 s (black arrow) for control‐, MRS2‐, and MRS2‐D216Q‐transfected cells. (b) Representative single exponential fits to the Mag‐Green fluorescence decays after the 3 mM MgCl_2_ addback shown in (a), reporting Mg^2+^ clearance and taken as a measure of mitochondrial Mg^2+^ uptake. (c) One‐way ANOVA followed by Tukey's post hoc comparison of mitochondrial Mg^2+^ uptake rates for control‐, MRS2‐, and MRS2‐D216Q‐transfected cells, where *p < 0.05, **p < 0.01, and ***p < 0.001 from *n* = 5 separate experiments. In (a–c), data were acquired at 22.5°C and were normalized as *F*/*F*_0_, where *F* is the Mag‐Green fluorescence at any time point and *F*_0_ is the mean 30 s baseline fluorescence recorded before the addition of EDTA/digitonin. (d) Representative fields of view of empty vector‐, MRS2‐ and MRS2‐D216Q‐overexpressing HeLa cell monolayers showing migration into the scratched area at 0, 24, 48, and 72 h postwound. Green scale bar is 1000 μm. (e) Time course of remaining wound area for empty vector‐, MRS2‐, and MRS2‐D216Q‐overexpressing HeLa cell monolayers shown as means ± SEM from *n* = 4 experiments. (f) One way ANOVA followed by Tukey's post hoc test comparing fraction of remaining wound area at 48 and 72 h, with **p < 0.01 and ***p < 0.001. (g) PI fluorescence emission spectra (means ± SEM of *n* = 5 experiments) reporting relative PI uptake, taken as a measure of 5 μM DXR‐induced cell death for control‐, MRS2‐, and MRS2‐D216Q‐transfected cells. (H) One‐way ANOVA followed by Tukey's post hoc comparison of relative PI fluorescence maximum for control‐, MRS2‐, and MRS2‐D216Q‐transfected cells, where **p < 0.01 and ***p < 0.001. In (a, b, c, f, g, and h), data from HeLa cells transfected with empty‐vector, MRS2, and MRS2‐D216Q are colored as gray, purple and yellow, respectively. In (g and h), blue shows data collected for empty‐vector transfected HeLa cells with no DXR treatment.

Having found that MRS2‐NTD‐D216Q is remarkably refractory to biophysical and structural changes induced by Mg^2+^, we next assessed how this mutation affected mitochondrial Mg^2+^ uptake. As we have previously done (Uthayabalan et al., 2023), we loaded HeLa cells over‐expressing full‐length MRS2, MRS2‐D216Q or an empty control vector with the cytosolic Mag‐Green Mg^2+^ indicator. To assess mitochondrial Mg^2+^ uptake, the PM was permeabilized with 5 μM digitonin, and 3 mM MgCl_2_ was introduced into the intracellular buffer (IB) bathing medium. Mitochondrial Mg^2+^ uptake rates were inferred from the clearance of extramitochondrial Mg^2+^, measured as the decrease in Mag‐Green fluorescence. After MgCl_2_ addback, the digitonin‐permeabilized cells transfected with empty pCMV vector (control), pBSD‐MRS2 (wild‐type), and pBSD‐MRS2‐D216Q (mutant), all displayed a decay in Mag‐Green fluorescence, associated with Mg^2+^ clearance (Figure 8a). Fitting the data to single exponential decays indicated greater extramitochondrial Mg^2+^ clearance rates for MRS2‐expressing cells compared with control cells; moreover MRS2‐D216Q‐expressing cells showed greater clearance rates compared to both control and MRS2‐expressing cells (Figure 8b,c). Thus, expression of MRS2‐D216Q leads to increased rates of Mg^2+^ uptake, consistent with the notion that the D216Q mutation abrogates Mg^2+^‐binding‐dependent autoinhibition of MRS2.

### 2.9 Overexpression of MRS2‐D216Q increases cell migration and resistance to apoptosis challenge

Given that the D216N was identified as a mutation in malignant melanoma tissue (Van Allen et al., 2015) and the robust loss in Mg^2+^‐sensitivity of MRS2‐NTD‐D216Q as well as markedly higher mitochondrial Mg^2+^ uptake rates of MRS2‐D216Q, suggestive of a deficiency in Mg^2+^ binding‐mediated autoinhibition, we next assessed two easily trackable hallmarks of cancer. First, we employed a monolayer scratch assay to evaluate cell migration as a measure of invasive potential (Liang et al., 2007). HeLa cells were grown on a 35 mm dish to 100% confluency post‐transfection with empty control, pBSD‐MRS2 or pBSD‐MRS2‐D216Q vectors. After applying a scratch across the center of the dish, migration was regularly quantified at 24, 48, and 72 h as the fraction of remaining wound area. Excitingly, cells overexpressing MRS2 showed significantly increased migration at 48 and 72 h; moreover, cells overexpressing MRS2‐D216Q, which showed higher mitochondrial Mg^2+^ uptake rates, also showed dramatically enhanced migration compared with both empty vector and wild‐type controls at 48 and 72 h (Figure 8d–f).

Doxorubicin (DXR) is used as cervical cancer treatment in the clinic and has been shown to induce apoptosis of cancer cells and associated lines including HeLa cells (Abdoul‐Azize et al., 2018; Jawad et al., 2019; Wang et al., 2022; Wei et al., 2020). Thus, as a second cancer hallmark assessment, we challenged HeLa cells with DXR and monitored cell death using propidium iodide (PI) fluorescence spectrophotometry. As previously shown, 5 μM DXR treatment for 5 h dramatically increased HeLa cell death (Merolle et al., 2018), as indicated by the higher PI fluorescence in empty‐vector transfected cells (Figure 8g,h). HeLa cells overexpressing MRS2 and undergoing a similar DXR treatment protocol showed decreased PI fluorescence (Figure 8g,h), consistent with past work showing MRS2 overexpression suppresses DXR‐induced apoptosis in HEK293 cells (Merolle et al., 2018). Excitingly, HeLa cells overexpressing MRS2‐D216Q showed markedly lower DXR‐induced apoptosis compared to wild‐type MRS2 overexpressing cells and empty‐vector control cells (Figure 8g,h). We also performed the experiment with 0.5 μM DXR but at longer incubation times (i.e., 48 h), finding a similar cell death pattern, where MRS2 overexpression reduced PI uptake and MRS2‐D216Q overexpression further potentiated this cell death protection (Figure S3A,B). Western blot analysis of empty‐, MRS2‐, or MRS2‐D216Q‐transfected cells showed the wild‐type MRS2 and MRS2‐D216Q were similarly and effectively overexpressed in HeLa cells compared with empty control‐transfected cells (Figure S3C–E).

Taken together with our biophysical analyses and mitochondrial Mg^2+^ uptake assessments, these data suggest that perturbation of Mg^2+^ binding to the MRS2‐NTD due to D216Q mutation increases mitochondrial Mg^2+^ uptake, which potentiates key hallmarks of cancer including cell migration and resistance to apoptosis.

## 3 DISCUSSION

Most if not all divalent cation channels have evolved feedback regulation mechanisms to prevent overloaded activities and cellular dysfunction. For example, inositol 1,4,5 trisphosphate receptors are negatively regulated by Ca^2+^ (Bezprozvanny et al., 1991) and other Ca^2+^ binding proteins (Li et al., 2013), ORAI1 channels undergo slow and fast Ca^2+^‐dependent inactivation (Parekh, 2017), ryanodine receptors sense numerous repressive inputs including Mg^2+^ and calmodulin binding (Meissner & Henderson, 1987) and the mitochondrial Ca^2+^ uniporter is negatively regulated by Ca^2+^ or Mg^2+^ binding to the matrix domain (Colussi & Stathopulos, 2023; Lee et al., 2016; Vais et al., 2020), to name a few. Indeed, we recently showed that Mg^2+^ binding to a site involving D216 and D220 on the MRS2‐NTD not only promoted domain disassembly, increased regular secondary structure, enhanced solvent exposed hydrophobicity and stabilized the domain, but also suppressed MRS2‐mediated mitochondrial Mg^2+^ uptake as negative feedback (Uthayabalan et al., 2023). Intriguingly, a malignant melanoma tissue sample was found to harbor a mutation at D216 of MRS2 (Van Allen et al., 2015). However, the very high conformational similarities of cryo‐EM structures with and without Mg^2+^ have provided limited information on Mg^2+^‐dependent MRS2 channel regulation (He et al., 2023; Lai et al., 2023; Li et al., 2023), particularly with respect to D216.

Thus, to gain more insight into the mechanisms of MRS2 regulation and function, we evaluated the effects of a D216Q mutation on MRS2‐NTD structure, biophysical properties, and function in the context Mg^2+^ uptake and hallmarks of cancer. Since our previous D216A/D220A substitution (Uthayabalan et al., 2023) and the D216N cancer‐associated mutation both decrease the overall propensity for α‐helicity of the DALVD sequence stretch, we specifically designed a D216Q substitution to perturb Mg^2+^ binding, while maintaining a wild‐type‐like MRS2‐NTD structure (Figure 1c). Using this approach, we minimized the possibility for confounding allosteric affects associated with the mutation and focused on the Mg^2+^ binding event. We find that MRS2‐NTD‐D216Q abrogates Mg^2+^‐mediated homodimer and higher order oligomer dissociation without the D220 mutation (Figures 2 and 4). We also demonstrate that Mg^2+^ decreases the MRS2‐NTD dimer association affinity by as much as ~100‐fold under the most dilute conditions tested, and this effect can be abrogated by the D216Q substitution (Figure 3, Figure S2, and Table 1). Consistent with this robust effect, the D216Q mutation alone was sufficient to preclude Mg^2+^ binding to MRS2‐NTD (Figure 5) as well as the increased α‐helicity and thermal stability exhibited in the presence of Mg^2+^ (Figure 6).

The highest resolution cryo‐EM structure of human MRS2 determined in the presence of 20 mM MgCl_2_ reveals four Mg^2+^ binding sites (Mg‐1, Mg‐2, Mg‐3, and Mg‐4; Figure 1b) (Li et al., 2023). Mg‐1 is located inside the pore mouth coordinated by the GMN motif (Li et al., 2023). Mg‐2 is located in the center of the matrix region of human MRS2 coordinated by the five D329 residues in the pentamer. Mg‐3 is located on the MRS2‐NTD, within the interface of two protomers, involving E138, E243, and D247 of subunit A and E312 of subunit B. Mg‐4 is further from the IMM and also occurs within the interface between two protomers (i.e., E297 and E293 from subunit A and E261 from subunit B; Figures 1b and 6a). Since Mg‐4 occurs across two subunits in the MRS2‐NTD and showed the shortest coordination distances among the cryo‐EM identified sites outside the pore region, implying the tightest binding, we assessed whether the E261Q mutant could affect the Mg^2+^ sensing properties we defined. MRS2‐NTD‐E261Q did not have any discernable effect on Mg^2+^‐dependent Cys‐crosslinking, monomerization, increased α‐helicity and associated increased stability (Figures 3 and 7). We note that a concurrent five‐site E257A/D260A/E261A/E262A/E263A mutation has been recently documented to enhance MRS2 activity (Ponnusamy et al., 2024) but the mechanistic basis for this observation is unclear given the multisite substitution.

Resistance to apoptosis is a hallmark of cancer, and overcoming apoptotic resistance remains a key objective of numerous therapeutic strategies, particularly in the context of MDR, which poses a significant barrier to effective chemotherapy in GC (Emran et al., 2022). A notable attribute of MDR is decreased intracellular drug accumulation, resulting from overexpression of ATP‐binding cassette (ABC) transporters (Choi, 2005; Sun et al., 2012). Heightened activity of these efflux pumps may coincide with the upregulation or increased activity of human MRS2 resulting in increased generation of Mg^2+^‐ATP. MRS2 is overexpressed in GC cells exhibiting the MDR phenotype, suggesting a potential role in promoting MDR by facilitating the Mg^2+^‐ATP requirement for ABC transporter function (Sodani et al., 2012). In line with these observations, we show that MRS2 overexpression increases mitochondrial Mg^2+^ uptake in HeLa cells. Further, the robust increase in extramitochondrial Mg^2+^ clearance rates for cells overexpressing the MRS2‐D216Q (Figure 8) is congruent with our data demonstrating D216Q effectively abrogates the structural and biophysical changes that occur to MRS2‐NTD in the presence of Mg^2+^ and the notion that MRS2‐NTD negative feedback regulation depends on Mg^2+^ binding involving D216.

Previous work showed that MRS2‐overexpressing HEK293 cells are more resistant to apoptosis induced by staurosporine and DXR (Merolle et al., 2018). Here, we show that HeLa cells overexpressing MRS2 also maintain higher viability after DXR treatment compared with control cells. Furthermore, consistent with the increased Mg^2+^ uptake mediated by MRS2‐D216Q due to limited negative feedback, cells expressing the Mg^2+^ binding mutant showed an even greater DXR‐induced apoptosis resistance than wild‐type MRS2 overexpressing cells (Figure 8). Cell migration was similarly affected, where HeLa cells overexpressing MRS2 showed enhanced migration but cells overexpressing MRS2‐D216Q showed even greater migration than MRS2‐overexpressing cells using simple scratch assays (Figure 8).

**FIGURE 9:**
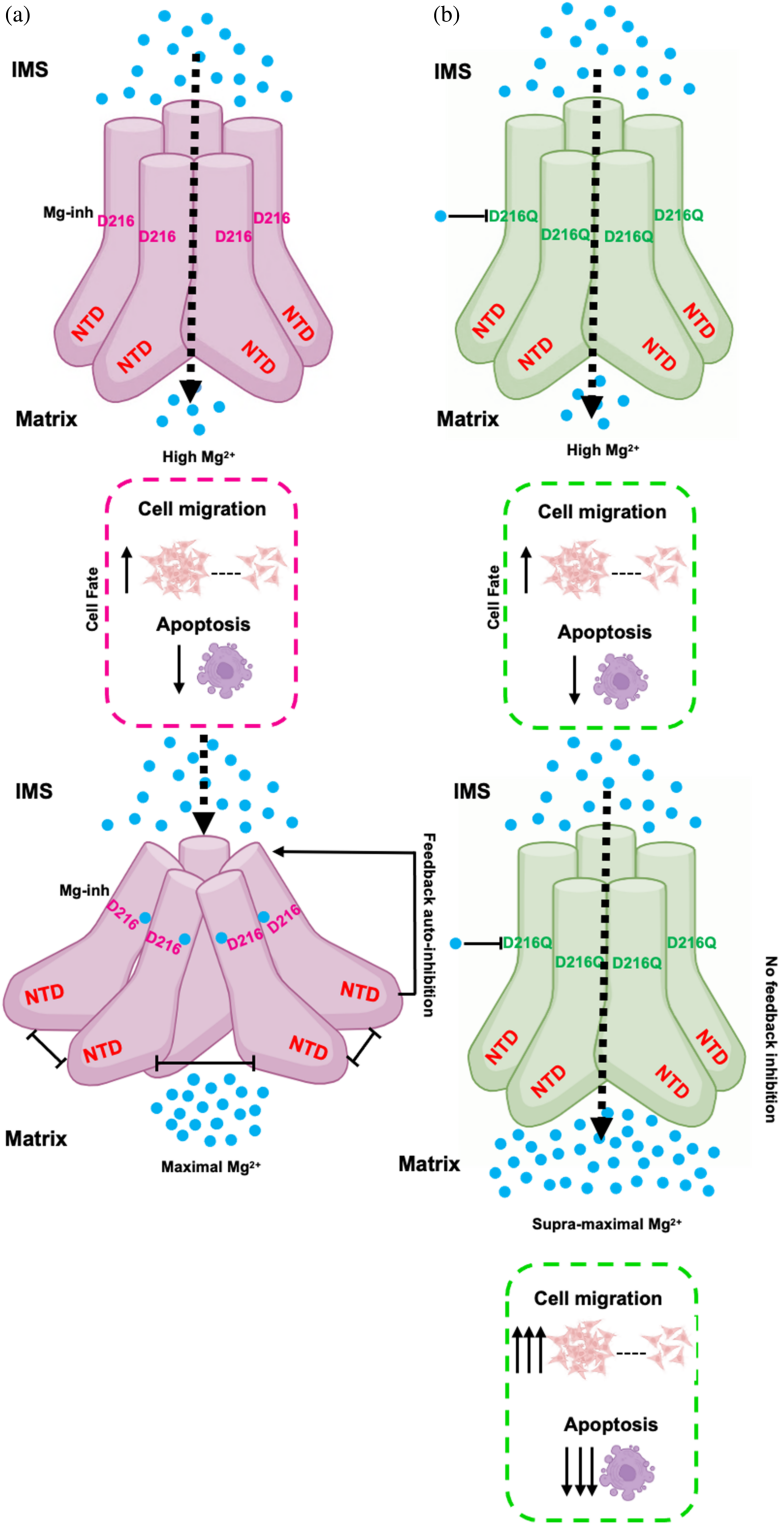
Model for D216‐dependent autoregulation and dysfunction of MRS2 activity. (a) High intermembrane space (IMS) Mg^2+^ activates wild‐type MRS2 (pink). When the inhibitory Mg^2+^ binding site (Mg‐inh) is not associated with Mg^2+^ (blue circle) the channel maintains maximal activity leading to increases in matrix Mg^2+^. Once matrix Mg^2+^ binds to Mg‐inh involving D216, NTD conformational changes robustly weaken the NTD:NTD interactions leading to feedback inhibition of the channel, thus protecting cells from Mg^2+^ overload and dysfunction. Nevertheless, abnormally high matrix Mg^2+^ from wild‐type MRS2 overexpression leads to increased cell migration and suppresses apoptosis. (b) High IMS Mg^2+^ activates MRS2‐D216Q (green) similar to wild‐type. However, mutations to D216 that prevent Mg^2+^ binding to Mg‐inh preclude the Mg^2+^‐dependent feedback inhibition, leading to supramaximal matrix Mg^2+^ and strongly potentiated cell migration and resistance to apoptosis.

Overall, the data presented here help define the human MRS2 negative feedback mechanism, whereby Mg^2+^ binding to the MRS2‐NTD involving D216 inhibits channel activity. Moreover, a simple D216Q mutation, designed to disrupt Mg^2+^ binding while maintaining wild‐type structure, alleviates this negative feedback and promotes supramaximal MRS2 activity. In pathophysiological scenarios where MRS2 is overexpressed, increased matrix Mg^2+^ mediated by MRS2 leads to apoptosis resistance and enhanced migration (Figure 9a). In scenarios involving mutations to the inhibitory Mg^2+^ binding site on the MRS2‐NTD (i.e., Mg‐inh), apoptosis resistance and migration are further enhanced (Figure 9b). The Mg^2+^‐dependent negative feedback is consistent with lower MRS2 orthologues where similar regulation mechanisms have been extensively defined (Chakrabarti et al., 2010; Dalmas et al., 2014; Eshaghi et al., 2006; Guskov et al., 2012; Johansen et al., 2022; Khan et al., 2013; Lunin et al., 2006; Payandeh & Pai, 2006; Pfoh et al., 2012).

## 4 MATERIALS AND METHODS

### 4.1 Expression and purification of MRS2_58_ _‐333_, MRS2 and mutants

MRS2_58‐333_ was expressed and purified using the approach we described previously (Uthayabalan et al., 2023). D216Q and E261Q mutants were introduced into MRS2_58‐333_ by PCR‐mediated site‐directed mutagenesis and expression and purification for these constructs were performed as described for MRS2_58‐333_. The complementary mutagenic primers were 5′‐CTGATCCTTGAGACCTTGCAAGCTTTGGTGGACCCC‐3′ and 3′‐GGGGTCCACCAAAGCTTGCAAGGTCTCAAGGATCAG‐5′ for D216Q and 5′‐GGAGATCTTGGATCAGGAAGAGTTGCTAGAAGAG‐3′ and 3′‐CTCTTCTAGCAACTCTTCCTGATCCAAGATCTCC‐5′ for E261Q.

### 4.2 Size exclusion chromatography with inline multiangle light scattering

SEC‐MALS analyses were conducted with a Superdex 200 Increase 10/300 GL column (Cytiva) connected to an AKTA Pure FPLC system (Cytiva). Molecular weight estimates for MRS2_58‐333_, MRS2_58‐333_‐D216Q and MRS2_58‐333_‐E261Q were obtained using a DAWN HELEOS II detector (Wyatt) and an Optilab TrEX differential refractometer (Wyatt). The entire inline FPLC/MALS system was housed in a cold cabinet maintained at ∼10°C. Data were acquired for proteins at a concentration of 2.5 mg/mL in 20 mM Tris (pH 8), 150 mM NaCl, and 1 mM DTT, with 100 μL injections of the sample at each concentration. MALS molecular weights were determined using Zimm plot analysis and a protein refractive index increment (dn/dc) of 0.185 L/g in the accompanying ASTRA software (version 7.1.4; Wyatt). Divalent cation‐containing experiments were conducted by supplementing running buffers and protein samples with 5 mM MgCl_2_.

### 4.3 Dynamic light scattering

DLS measurements were made with a DynaPro NanoStar (Wyatt) instrument using a scattering angle of 90°. After centrifugation at 15,000*g* for 10 min at 4°C, 5 μL of supernatant was loaded into a JC‐501 microcuvette, and measurements were collected as 10 consecutive acquisition scans with each acquisition being an average of 5 s. MRS2_58‐333_ and MRS2_58‐333_‐D216Q protein samples were assessed at 1.25 mg/mL in 20 mM Tris (pH 8), 150 mM NaCl, and 1 mM DTT in the absence or presence of 5 mM MgCl_2_. For both proteins, data were acquired at 20°C. All autocorrelation functions were deconvoluted using the regularization algorithm to extract the polydisperse distribution of hydrodynamic radii (*R*_h_) using the accompanying Dynamics software (version 7.8.1.3; Wyatt).

### 4.4 Intrinsic fluorescence measurement for cation binding

A Cary Eclipse spectrofluorimeter (Agilent/Varian) was used to acquire intrinsic fluorescence emission spectra. Spectra were acquired for 0.1 mg/mL MRS2_58‐333_ and MRS2_58‐333_‐D216Q in 20 mM Tris (pH 8), 150 mM NaCl, and 1 mM DTT, using a 600‐μL quartz cuvette. The fluorescence emission intensities were recorded at 22.5°C from 300 to 450 nm, using a 1 nm data pitch and an excitation wavelength of 280 nm. Excitation and emission slit widths were set to 5 and 10 nm, respectively, and the photomultiplier tube detector was set to 650 V. Emission spectra were obtained before and after supplementation with increasing concentrations MgCl_2_ added directly to the cuvette. A total of 15 emission spectra were acquired with increasing concentrations of divalent cation between 0 and 5 mM. Spectral intensities at 350 nm were corrected for the dilution associated with the volume change upon each addition to the cuvette, and resultant curves were fit to a one site binding model that considers protein concentration using R (version 4.2.1) to extract apparent equilibrium dissociation constants (*K*_d_).

### 4.5 Far‐UV CD spectroscopy

Far‐UV CD spectra were acquired using a Jasco J‐810 CD spectrometer with electronic Peltier temperature regulator (Jasco). Each spectrum was taken as an average of three accumulations, recorded at 37°C using a 1‐mm path length quartz cuvette in 1‐nm increments, 8‐s averaging time, and 1 nm bandwidth. To eliminate technical variability in magnitude signals, after acquiring divalent cation‐free spectra, 5 mM MgCl_2_ was added to the same samples, and spectra were re‐acquired. Thermal melts were recorded using a 1‐mm path length quartz cuvette by monitoring the change in the CD signal at 222 nm from 20 to 70°C. A scan rate of 1°C min^−1^, 1 nm bandwidth, and 8‐s averaging time was used during data acquisition. Mg^2+^‐free and Mg^2+^‐supplemented data were fit using a Boltzmann sigmoidal equation to estimate the midpoint of temperature denaturation (*T*_m_) using R (version 4.2.1). The protein concentration was 0.5 mg/mL and the buffer was 20 mM Tris, 150 mM NaCl, 1 mM DTT, pH 8.0.

### 4.6 Analytical ultracentrifugation

Sedimentation equilibrium studies were carried out using a Beckman Optima XL‐A Analytical Ultracentrifuge (Beckman) at 20°C. An An60Ti rotor and six‐channel cells with Epon‐charcoal centerpieces were used for the data acquisition. Absorbance measurements at 297 nm were collected in 0.002 cm radial steps and averaged over 10 readings. Final dialyzed MRS2_58‐333_ and MRS2_58‐333_‐D216Q were concentrated to a minimum of 2.5 mg/mL. AUC was performed with three concentrations (1.25, 0.625, and 0.312 mg/mL) in both Mg^2+^‐free and Mg^2+^‐loaded states. For loading, 110 μL of the sample buffers and 100 μL of samples with different protein concentrations were pipetted into the channel centerpieces. Data were analyzed using the global single ideal species model and according to a monomer‐dimer equilibrium model. The experimental buffer was 20 mM Tris, 150 mM NaCl, 1 mM DTT, pH 8.0.

### 4.7 Cysteine cross‐linking

MRS2_58‐333_, MRS2_58‐333_‐D216Q, and MRS2_58‐333_‐E261Q protein samples (0.25 mg/mL) were cross linked using 3.2 mM diamide and allowed to rest at 4°C for 5 h. Samples were subsequently separated on a 15% (wt/vol) SDS‐PAGE gel, and protein bands were visualized by Coomassie blue staining. Data are representative of *n* = 3 separate experiments and were completed in 20 mM Tris and 150 mM NaCl, pH 8.0.

### 4.8 Mitochondrial Mg^2+^ uptake assays using Mag‐Green

HeLa cells were cultured in DMEM with high glucose (Wisent), 10% (vol/vol) FBS (Sigma‐Aldrich), 100 μg/mL penicillin, and 100 U/mL streptomycin (Wisent) at 37°C in a 5% CO_2_, 95% (vol/vol) air mixture. Cells cultured in 35‐mm dishes were transfected with PolyJet transfection reagent (SignaGen) according to the manufacturer guidelines. After ∼12 h, cells were incubated with 0.725 μM of the Mg^2+^ indicator Mag‐Green for 30 min at 37°C. Cells were subsequently washed in divalent cation‐free phosphate buffered saline (PBS), pH 7.4, and suspended in 2 mL of IB composed of 20 mM HEPES (pH 7), 130 mM KCl, 2 mM KH_2_PO_4_, 10 mM NaCl, 5 mM succinate, 5 mM malate, and 1 mM pyruvate. A 20% (vol/vol) cell suspension in IB was created in a quartz cuvette. Mag‐Green fluorescence was monitored using a PTI QuantMaster spectrofluorimeter (Horiba) equipped with electronic temperature control using excitation and emission wavelengths of 506 and 531 nm, respectively, and excitation and emission slit widths of 2.5 and 2.5 nm, respectively. After 30 s Mag‐Green baseline fluorescence measurement, 2 mM EDTA plus 5 μM digitonin was added to permeabilize the plasma membrane. After 300 s, 3 mM MgCl_2_ was added to the cuvette and the Mag‐Green signal was measured for 500 s. The first three intensity values recorded immediately after the cation addback were not included in any trace because of potential mixing and light scattering artifact contributions. Mitochondrial Mg^2+^ uptake was correlated with the clearance of Mg^2+^, taken as the decrease in Mag‐Green fluorescence after re‐introduction of Mg^2+^ into the system, as previously done (Daw et al., 2020; Uthayabalan et al., 2023). The rates of Mag‐Green fluorescence decrease were extracted by fitting the traces after the Mg^2+^ addback to a single exponential decay in R (version 4.2.1).

### 4.9 Scratch migration assays

HeLa cells were cultured in DMEM with high glucose (Wisent), 10% (vol/vol) FBS (Sigma‐Aldrich), 100 μg/mL penicillin, and 100 U/mL streptomycin (Wisent) at 37°C in a 5% CO_2_, 95% (vol/vol) air mixture. Upon reaching 80% confluence, cells cultured in 35 mm dishes were transfected with PolyJet transfection reagent (SignaGen) according to the manufacturer guidelines. Cells were grown to 100% confluency, after which, a P200 pipette tip was used to scratch a trench across the coverslip. Cells were imaged at 0, 24, 48, and 72 h post‐scratch with a Zeiss AXIO Observer.D1 Inverted Fluorescence Microscope (Zeiss) using a ×10 objective lens. ImageJ (version 1.8.0) measured the proportion of the scratch width that diminished from cells migrating into the scratch area.

### 4.10 Apoptosis assay using doxorubicin

Post‐transfection, cells were treated with 5 μM Doxorubicin for 5 h or 0.5 μM Doxorubicin for 48 h, followed by a 5 min incubation with 5 μM PI. Subsequently, cells were washed and suspended in divalent cation‐free PBS (pH 7.4) within a quartz cuvette. Propidium iodide fluorescence was monitored using a PTI QuantMaster spectrofluorimeter (Horiba) equipped with electronic temperature control, using excitation wavelength of 550 nm and acquiring an emission spectrum between 560 and 650 nm. Excitation and emission slit widths were set to 20 and 10 nm, respectively. After a 15 min temperature equilibration at 22.5°C, the PI emission spectrum was measured. A volume of 50 μL of cells was deposited on a hemacytometer and counted using a Zeiss Axiovert 40 C Inverted Phase Contrast Microscope with a ×40 objective lens.

### 4.11 Western blot

Twenty‐four hours post‐transfection performed as described above, cells were harvested using a scraper, washed twice with ice‐cold PBS and proteins were solubilized by incubation with RIPA buffer (150 mM NaCl, 1% [wt/vol] Triton X‐100, 0.5% [wt/vol] sodium deoxycholate, 0.1% [wt/vol] SDS, 50 mM Tris [pH 7.5], and 1 mL of protease inhibitor cocktail [Millipore‐Sigma]) for 15 min. After centrifugation at 15,000*g* for 15 min at 4°C, the supernatants were collected, and protein concentrations were determined using a bicinchoninic acid (BCA) assay (Pierce BCA Protein Assay Kit, Thermo Scientific). Equal amounts of protein were separated on a 15% (wt/vol) SDS‐PAGE gel. Proteins were subsequently transferred to a PVDF membrane (Millipore) using a semi‐dry transfer system (Bio‐Rad). Following transfer, the PVDF membrane was blocked in TBST containing 3% (wt/vol) non‐fat dry milk powder for 2 h with gentle shaking at room temperature. After blocking, the membrane was washed with TBST, and incubated with polyclonal rabbit anti‐human MRS2 (Invitrogen, PA5‐553816) diluted 1:1250 in TBST supplemented with 1% (wt/vol) BSA for 1 h at room temperature with gentle shaking. After washing with TBST, the membrane was incubated with HRP‐conjugated goat anti‐rabbit IgG (Cell Signaling Technology, 7074) diluted 1:1250 in TBST supplemented with 1% (wt/vol) BSA for 1 h at room temperature with gentle shaking. After washing with TBST, the membrane was incubated with enhanced chemiluminescence substrate for 2 min and imaged using a ChemiDoc MP Imaging System (Bio‐Rad). Signal intensities were quantified using Image Lab software (Bio‐Rad) to quantify protein expression levels.

After stripping the blot with buffer containing 200 mM glycine, 0.1% (wt/vol) SDS, 1% tween‐20, pH 2.2 for 15 min, the membrane was washed and incubated with recombinant rabbit polyclonal anti‐human GAPDH (Millipore‐Sigma, ZB374) diluted 1:2500 in TBST supplemented with 1% (wt/vol) BSA for 1 h at room temperature with gentle shaking. Washing, secondary antibody incubation, detection, imaging and analysis was performed as described for MRS2.

### 4.12 Statistics

Unpaired *t*‐test was used when comparing two independent groups, paired *t*‐test was used when comparing outcomes of the same group before and after treatment, and one‐way ANOVA followed by Tukey's post hoc test was used for multiple means comparisons between three or more groups. All nonlinear regression fitting and statistical analyses were done in GraphPad Prism (4.03) or R (4.2.1).

## AUTHOR CONTRIBUTIONS

**Sukanthathulse Uthayabalan:** Conceptualization; investigation; writing – original draft; methodology; visualization; writing – review and editing; formal analysis; validation. **Taylor Lake:** Methodology. **Peter B. Stathopulos:** Conceptualization; funding acquisition; writing – review and editing; resources; supervision; data curation; project administration.

## Supporting information


**Figure S1.** Sequence logo showing conservation of MRS2 family proteins. (A) Sequence logo of MRS2 orthologues. Sequence logo output was derived from WebLogo from the Clustal Omega alignment of sequences for *Homo sapiens* (NCBI Reference Sequence: NP_065713.1), *Bos taurus* (NP_001095373.1), *Canis lupus* (XP_038302408.1), *Oryctolagus cuniculus (*XP_002714204.2), *Mus musculus* (NP_001013407.2), *Gallus gallus* (XP_040519067.1), *Ornithorhynchus anatinus* (XP_028909352.1), *Zootoca vivipara* (XP_060133926.1), *Rana temporaria* (XP_040209734.1), *Danio rerio* (XP_693621.5), using default settings (Sievers et al., 2011). The x‐axis shows mapping of single nucleotide polymorphisms (SNPs) for human MRS2 in accordance with gnomAD v2.1.1, and the y‐axis displays the maximum entropy for the given residue position. The height of each letter is proportional to the conservation of the residue at that position. Acidic residues [DE] are red; basic [HKR] are blue; hydrophobic [ACFILMPVW] are black; neutral [GNQSTY] are green. Below the sequence logo, the (*) indicates missense mutations; (=) silent mutations, and (:) predicted loss of function variants. Residue ranges corresponding to the Mg^2+^ binding sites shown in Figure 1b are boxed with the corresponding color.

**Figure S2.** Global single ideal molecular weight and monomer‐dimer K_d_ fits of MRS2_58‐333_ and MRS2_58‐333_‐D216Q in the absence and presence of MgCl_2_. Sedimentation equilibrium measurements of MRS2_58‐333_ at (A) 1.25 mg/mL, (B) 0.625 mg/mL and **(C)** 0.312 mg/mL in the absence of Mg^2+^. Sedimentation equilibrium of MRS2_58‐333_ at **(D)** 1.25 mg/mL, **(E)** 0.625 mg/mL and **(F)** 0.312 mg/mL in the presence of 5 mM MgCl_2_. Sedimentation equilibrium of MRS2_58‐333_‐D216Q at **(G)** 1.25 mg/mL **(H)**, 0.625 mg/mL and **(I)** 0.312 mg/mL in the presence of 5 mM MgCl_2_. Data were acquired at 8000, 12,000, 16,000 and 20,000 rpm. The red lines through the data in (*A‐I*) are global single ideal molecular weight fits within each respective panel. The residuals for the fits are shown on the top of each panel. Globally fitted monomer‐dimer equilibrium dissociation constants (K_d_) showed residuals nearly identical to those observed for the single ideal molecular weight fits (not shown). Data in (*A‐I*) were acquired in 20 mM TRIS, 150 mM NaCl, 1 mM DTT, pH 8 at 25°C.

**Figure S3.** Low DXR concentration (0.5 μM)‐induced cell death of HeLa cells and western blot showing similar MRS2 and MRS2‐D216Q overexpression levels. (A) PI fluorescence emission spectra (means ± SEM of n = 5 experiments) reporting relative PI uptake, taken as a measure of cell death for control‐, MRS2‐, and MRS2‐D216Q ‐transfected cells. **(B)** One‐way ANOVA followed by Tukey's post hoc comparison of relative PI fluorescence maximum for control‐, MRS2‐, and MRS2‐D216Q‐transfected cells, where **P < 0.01 and ***P < 0.001. In (*A and B*), data from HeLa cells transfected with empty‐vector, MRS2 and MRS2‐D216Q are colored as gray, purple and yellow, respectively, while data collected for empty‐vector transfected HeLa cells with no DXR treatment are colored blue. **(C)** Western blot showing protein expression levels in empty vector‐, MRS2‐ and MRS2‐D216Q ‐transfected cells. Experimental groups are indicated at top and ladder (L) molecular weights (MW) at left of the blots. Each lane represents a separate transfection. MRS2‐MYC‐FLAG [MW: 47.9 kDa excluding the mitochondrial targeting sequence (MTS)] migrates slightly higher than endogenous MRS2 (MW: 44.3 kDa excluding the MTS) due to the presence of 31 additional amino acids from MYC‐tag, FLAG‐tag and linkers. **(D)** The blot in (*C*) was stripped and re‐probed for GAPDH (MW: 36.1 kDa). The band above the main GAPDH band, running close to 48 kDa, is residual anti‐MRS2 that was not fully stripped. **(E)** One‐way ANOVA followed by Tukey's post hoc comparison of relative MRS2/GAPDH intensity ratios for empty‐, MRS2‐, and MRS2‐D216Q‐transfected cells, where **P < 0.01 and ***P < 0.001 from n = 3 separate transfections for each group.

**Table S1:** Summary of SEC‐MALS and thermal stability data for MRS2_58‐333_, MRS2_58‐333_‐D216Q and MRS2_58‐333_‐E216Q proteins.
**Table S2:** Summary of the fitted Mg^2+^ binding equilibrium dissociation constants (K_d_).
