# Supplementary figures and images for: MRS2 missense variation at Asp216 abrogates inhibitory Mg2+ binding, potentiating cell migration and apoptosis resistance
Source: Protein Sci. 2024 Jul 11;33(8):e5108. doi: 10.1002/pro.5108 (PMC11237551; doi:10.1002/pro.5108)

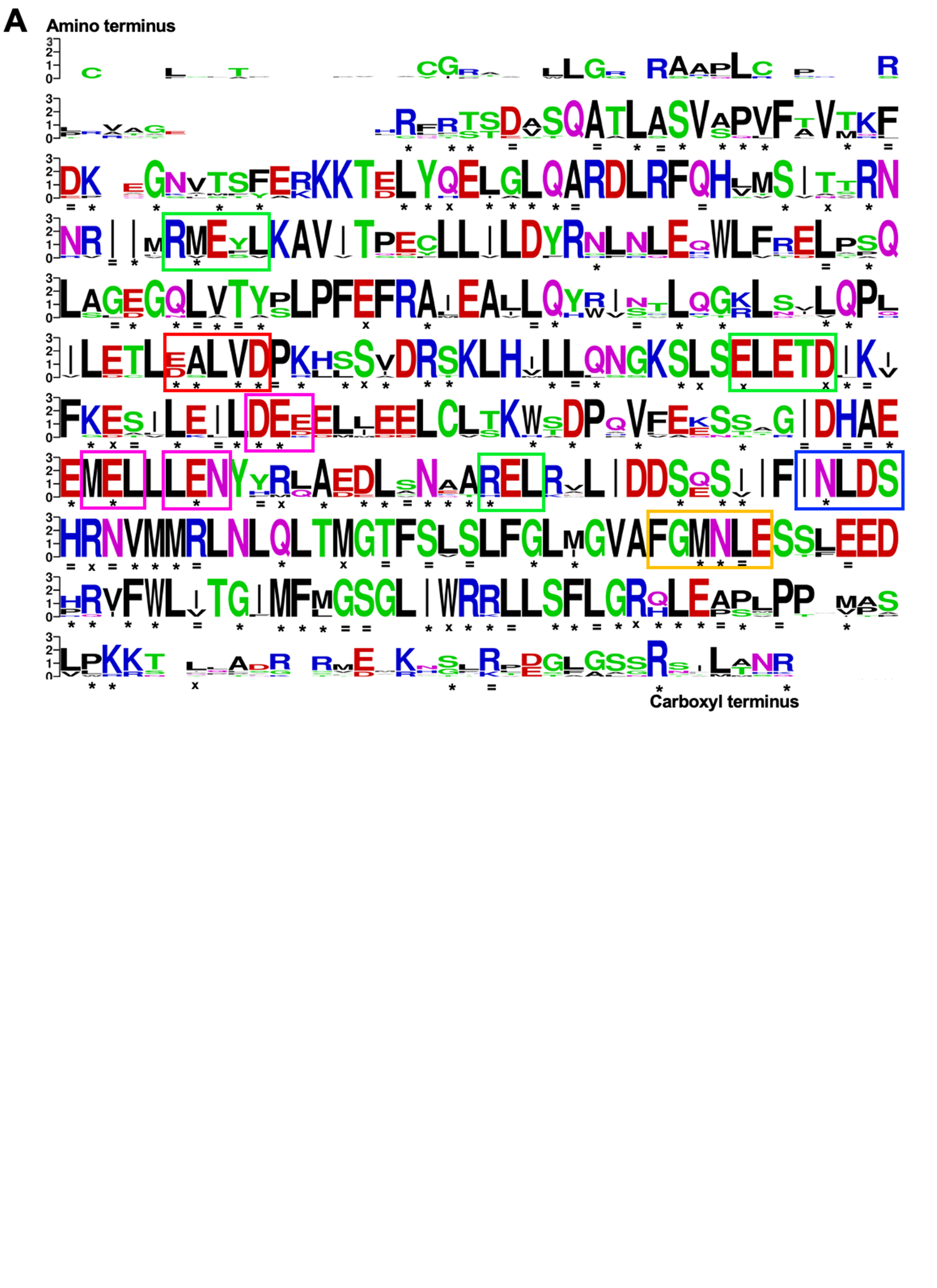

Supplement: Supplementary file 1 — Figure S1. Sequence logo showing conservation of MRS2 family proteins. (A) Sequence logo of MRS2 orthologues. Sequence logo output was derived from WebLogo from the Clustal Omega alignment of sequences for Homo sapiens (NCBI Reference Sequence: NP_065713.1), Bos taurus (NP_001095373.1), Canis lupus (XP_038302408.1), Oryctolagus cuniculus (XP_002714204.2), Mus musculus (NP_001013407.2), Gallus gallus (XP_040519067.1), Ornithorhynchus anatinus (XP_028909352.1), Zootoca vivipara (XP_060133926.1), Rana temporaria (XP_040209734.1), Danio rerio (XP_693621.5), using default settings (Sievers et al., 2011). The x‐axis shows mapping of single nucleotide polymorphisms (SNPs) for human MRS2 in accordance with gnomAD v2.1.1, and the y‐axis displays the maximum entropy for the given residue position. The height of each letter is proportional to the conservation of the residue at that position. Acidic residues [DE] are red; basic [HKR] are blue; hydrophobic [ACFILMPVW] are black; neutral [GNQSTY] are green. Below the sequence logo, the (*) indicates missense mutations; (=) silent mutations, and (:) predicted loss of function variants. Residue ranges corresponding to the Mg2+ binding sites shown in Figure 1b are boxed with the corresponding color. [file PRO-33-e5108-s003.TIF]

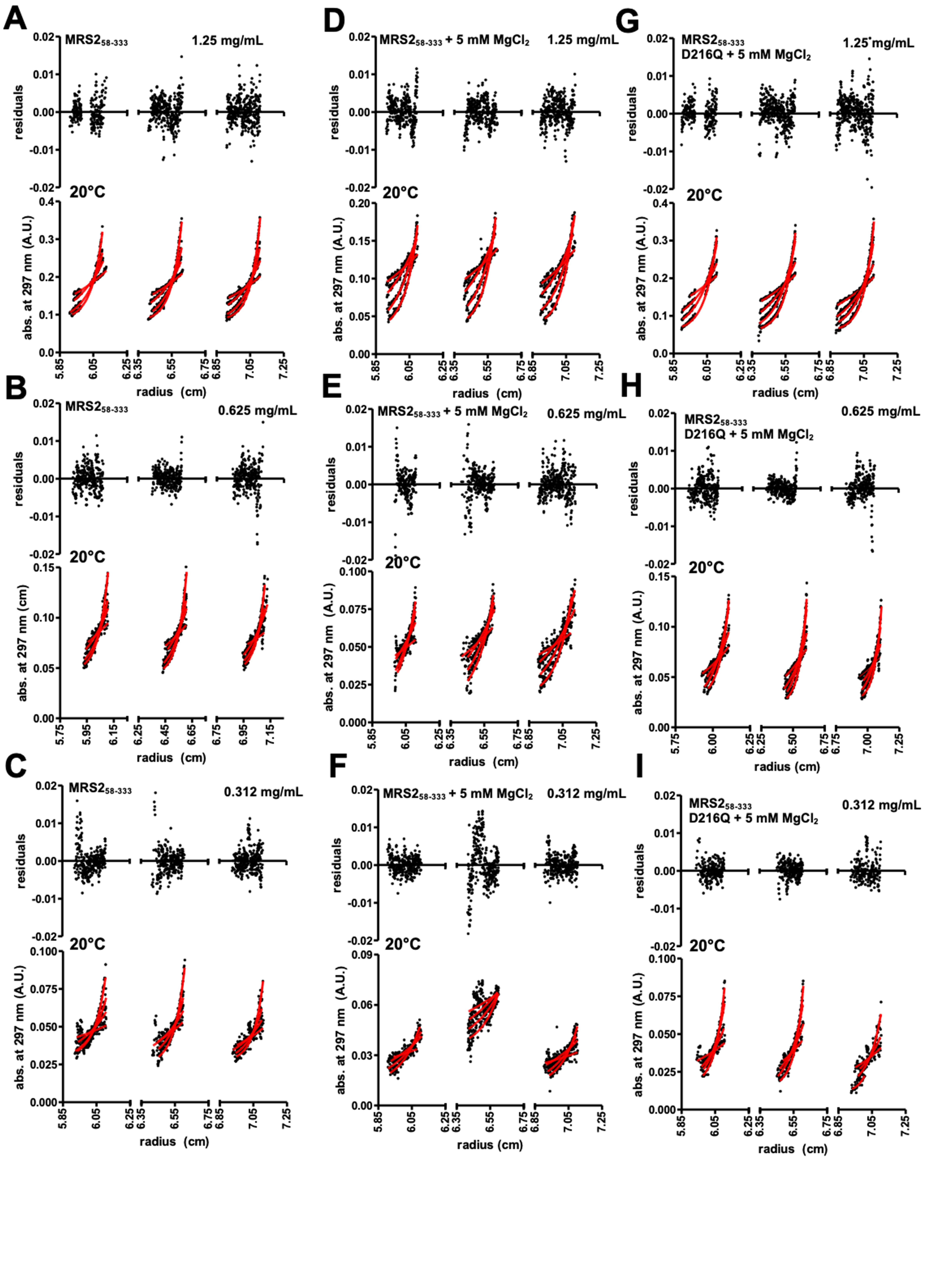

Supplement: Supplementary file 2 — Figure S2. Global single ideal molecular weight and monomer‐dimer Kd fits of MRS258‐333 and MRS258‐333‐D216Q in the absence and presence of MgCl2. Sedimentation equilibrium measurements of MRS258‐333 at (A) 1.25 mg/mL, (B) 0.625 mg/mL and (C) 0.312 mg/mL in the absence of Mg2+. Sedimentation equilibrium of MRS258‐333 at (D) 1.25 mg/mL, (E) 0.625 mg/mL and (F) 0.312 mg/mL in the presence of 5 mM MgCl2. Sedimentation equilibrium of MRS258‐333‐D216Q at (G) 1.25 mg/mL (H), 0.625 mg/mL and (I) 0.312 mg/mL in the presence of 5 mM MgCl2. Data were acquired at 8000, 12,000, 16,000 and 20,000 rpm. The red lines through the data in (A‐I) are global single ideal molecular weight fits within each respective panel. The residuals for the fits are shown on the top of each panel. Globally fitted monomer‐dimer equilibrium dissociation constants (Kd) showed residuals nearly identical to those observed for the single ideal molecular weight fits (not shown). Data in (A‐I) were acquired in 20 mM TRIS, 150 mM NaCl, 1 mM DTT, pH 8 at 25°C. [file PRO-33-e5108-s004.TIF]

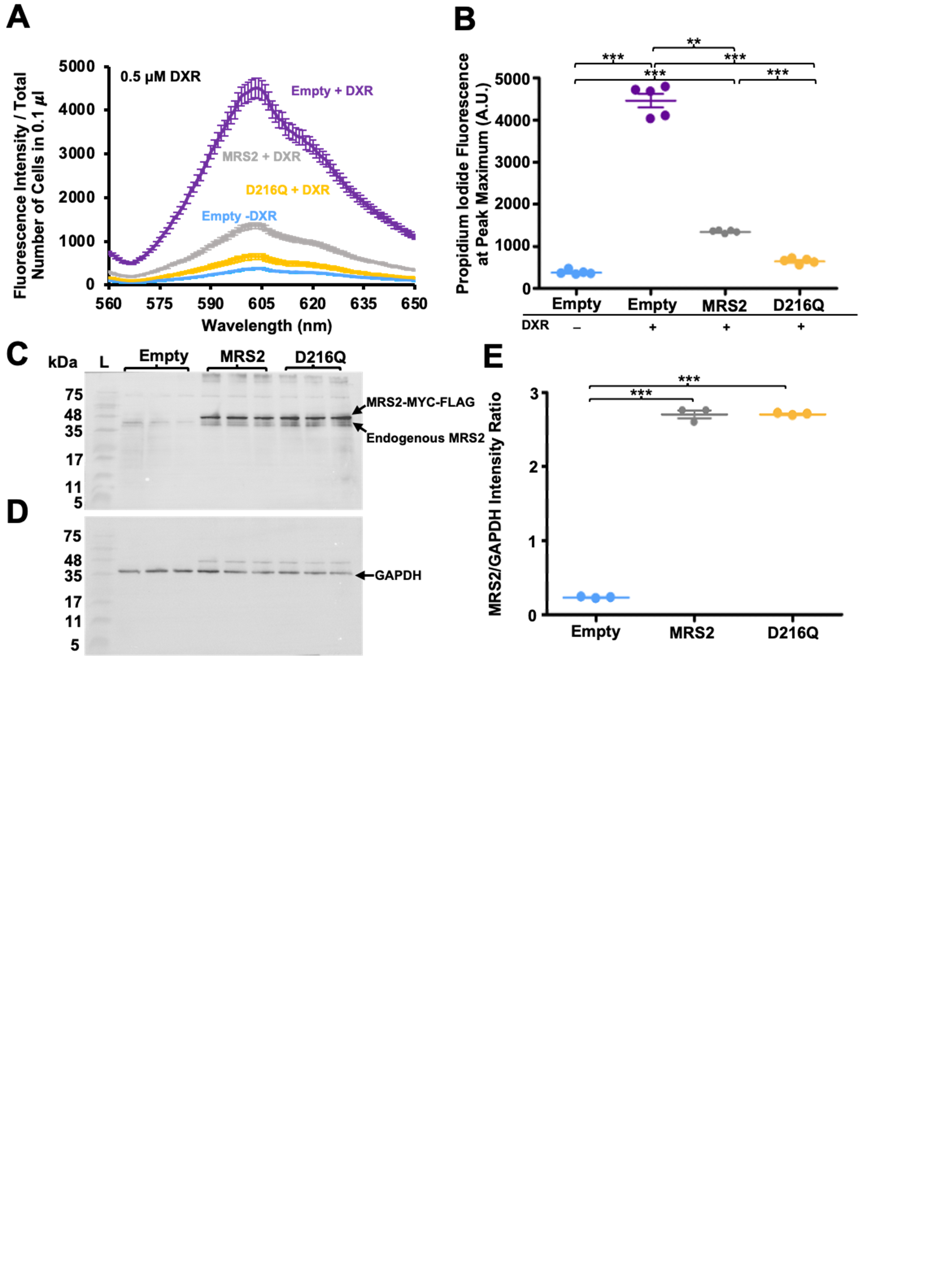

Supplement: Supplementary file 3 — Figure S3. Low DXR concentration (0.5 μM)‐induced cell death of HeLa cells and western blot showing similar MRS2 and MRS2‐D216Q overexpression levels. (A) PI fluorescence emission spectra (means ± SEM of n = 5 experiments) reporting relative PI uptake, taken as a measure of cell death for control‐, MRS2‐, and MRS2‐D216Q ‐transfected cells. (B) One‐way ANOVA followed by Tukey's post hoc comparison of relative PI fluorescence maximum for control‐, MRS2‐, and MRS2‐D216Q‐transfected cells, where **P < 0.01 and ***P < 0.001. In (A and B), data from HeLa cells transfected with empty‐vector, MRS2 and MRS2‐D216Q are colored as gray, purple and yellow, respectively, while data collected for empty‐vector transfected HeLa cells with no DXR treatment are colored blue. (C) Western blot showing protein expression levels in empty vector‐, MRS2‐ and MRS2‐D216Q ‐transfected cells. Experimental groups are indicated at top and ladder (L) molecular weights (MW) at left of the blots. Each lane represents a separate transfection. MRS2‐MYC‐FLAG [MW: 47.9 kDa excluding the mitochondrial targeting sequence (MTS)] migrates slightly higher than endogenous MRS2 (MW: 44.3 kDa excluding the MTS) due to the presence of 31 additional amino acids from MYC‐tag, FLAG‐tag and linkers. (D) The blot in (C) was stripped and re‐probed for GAPDH (MW: 36.1 kDa). The band above the main GAPDH band, running close to 48 kDa, is residual anti‐MRS2 that was not fully stripped. (E) One‐way ANOVA followed by Tukey's post hoc comparison of relative MRS2/GAPDH intensity ratios for empty‐, MRS2‐, and MRS2‐D216Q‐transfected cells, where **P < 0.01 and ***P < 0.001 from n = 3 separate transfections for each group. [file PRO-33-e5108-s002.TIF]
