# Supplementary material for: MRS2 missense variation at Asp216 abrogates inhibitory Mg2+ binding, potentiating cell migration and apoptosis resistance
Source: Protein Sci. 2024 Jul 11;33(8):e5108. doi: 10.1002/pro.5108 (PMC11237551; doi:10.1002/pro.5108)
Supplement: Supplementary file 4 — Table S1: Summary of SEC‐MALS and thermal stability data for MRS258‐333, MRS258‐333‐D216Q and MRS258‐333‐E216Q proteins. Table S2: Summary of the fitted Mg2+ binding equilibrium dissociation constants (Kd). [file PRO-33-e5108-s001.docx]

**Table S1: Summary of SEC-MALS and thermal stability data for MRS2_58–333_, MRS2_58-333_-D216Q and MRS2_58-333_-E216Q.**

| **Protein**  **(MRS2_58-333_)** | **Protein concentration (mg/mL)** | **MgCl_2_**  **Concentration**  **(mM)** | **Molecular Weight**  **(kDa) ^a^** | **Stoichiometric**  **Ratio ^b^** | **Elution Volume (mL)** | **T_m_ (°C)** |  |
| --- | --- | --- | --- | --- | --- | --- | --- |
| **WT** | 2.5 | 0 | 59.4 ± 3.7 | 1.84 | 14.4 | 51.9 |  |
|  |  | 0 | 53.5 ± 1.9 | 1.66 | 15.4 | 52.2 |  |
|  |  | 0 | 59.9 ± 1.5 | 1.86 | 14.0 | 51.3 |  |
|  |  | **Average:** | **57.6 ± 2.4** | **1.78 ± 0.63** | **14.7 ± 0.72** | **51.5 ± 0.45** |  |
|  |  | 5 | 33.5 ± 1.8 | 1.10 | 16.9 | 57.4 |  |
|  |  | 5 | 38.5 ± 1.8 | 1.19 | 16.5 | 57.5 |  |
|  |  | 5 | 28.9 ± 3.3 | 0.89 | 17.4 | 57.5 |  |
|  |  | **Average:** | **34.0 ± 2.3** | **1.06 ± 0.08** | **17.07 ± 0.45** | **57.5 ± 0.03** |  |
| **D216Q** | 2.5 | 0 | 58.2 ± 6.2 | 1.80 | 14.7 | 51.6 |  |
|  |  | 0 | 53.1 ± 2.7 | 1.64 | 15.5 | 51.5 |  |
|  |  | 0 | 54.0 ± 2.5 | 1.69 | 15.0 | 51.9 |  |
|  |  | **Average:** | **55.1 ± 3.8** | **1.71 ± 0.04** | **15.0 ± 0.45** | **51.7 ± 0.12** |  |
|  |  | 5 | 55.9 ± 1.4 | 1.86 | 15.2 | 52.9 |  |
|  |  | 5 | 53.5 ± 1.5 | 1.66 | 15.3 | 51.1 |  |
|  |  | 5 | 57.0 ± 2.4 | 1.77 | 14.9 | 52.7 |  |
|  |  | **Average:** | **55.5 ± 1.8** | **1.76 ± 0.05** | **15.1 ± 0.20** | **52.2 ± 0.56** |  |
| **E261Q** | | 2.5 | 0 | 56.4 ± 1.7 | 1.75 | 15.2 | 52.2 |
|  |  |  | 0 | 60.4 ± 4.6 | 1.87 | 14.0 | 52.2 |
|  |  |  | 0 | 55.2 ± 2.4 | 1.71 | 15.1 | 51.4 |
|  |  |  | **Average:** | **57.3 ± 2.9** | **1.77 ± 0.05** | **14.7 ± 0.66** | **51.9 ± 0.26** |
|  |  |  | 5 | 35.3 ± 4.8 | 1.09 | 16.7 | 56.8 |
|  |  |  | 5 | 28.3 ± 1.4 | 0.86 | 17.5 | 56.1 |
|  |  |  | 5 | 32.8 ± 3.9 | 1.12 | 17.1 | 57.1 |
|  |  |  | **Average:** | **31.8 ± 3.1** | **1.02 0.08** | **17.1 ± 0.23** | **56.6 ± 0.29** |

^a^ SEC-MALS-determined molecular weight.

^b^ Stoichiometric ratio was calculated as the SEC-MALS-determined molecular weight divided by the theoretical monomeric molecular weight of MRS2_58-333_ (32.2 kDa).

**Table S2: Summary of the fitted Mg^2+^ binding equilibrium dissociation constants (K_d_).**

| **Protein**  **(MRS2_58-333_)** | **Cation** | **K_d_ (mM) ^a^** | **Error ^b^** | **Avg. K_d_ (mM) ^c^** |
| --- | --- | --- | --- | --- |
| **WT** | Mg^2+^ | 0.132 | ± 0.030 | 0.13 ± 0.04 |
|  |  | 0.126 | ± 0.059 |  |
|  |  | 0.139 | ± 0.044 |  |
| **D216Q** | Mg^2+^ | 11.349 | ± 12.948 | 11.95 ± 10.05 |
|  |  | 11.324 | ± 9.723 |  |
|  |  | 13.191 | ± 7.494 |  |

^a^ Data are extracted from fits to a one-site binding model that accounts for protein concentration.

^b^ Errors (±) are fitted errors from the one-site binding model fit.

^c^ Errors (±) are SEM from the n=3 K_d_ values reported, determined using three protein preparations.
